# Deciphering the Microbiota and Volatile Profiles of Algerian *Smen*, a Traditional Fermented Butter

**DOI:** 10.3390/microorganisms10040736

**Published:** 2022-03-29

**Authors:** Rania Boussekine, Farida Bekhouche, Stella Debaets, Anne Thierry, Marie-Bernadette Maillard, Hélène Falentin, Audrey Pawtowski, Malika Barkat, Monika Coton, Jérôme Mounier

**Affiliations:** 1 Laboratoire de Biotechnologie et Qualité des Aliments (BIOQUAL), Institut de la Nutrition, de l’Alimentation et des Technologies Agro-Alimentaires (INATAA), Université Frères Mentouri Constantine 1, Route de Ain-El-Bey, Constantine 25000, Algeria; rania.boussekine@umc.edu.dz (R.B.); bekhouche.farida@umc.edu.dz (F.B.); barkat.malika@umc.edu.dz (M.B.); 2 Laboratoire Universitaire de Biodiversité et Ecologie Microbienne, INRAE, Univ Brest, F-29280 Plouzané, France; stella.debaets@univ-brest.fr (S.D.); audrey.pawtowski@univ-brest.fr (A.P.); monika.coton@univ-brest.fr (M.C.); 3 STLO, Institut Agro, INRAE, F-35000 Rennes, France; anne.thierry@inrae.fr (A.T.); marie-bernadette.maillard@inrae.fr (M.-B.M.); helene.falentin@inrae.fr (H.F.)

**Keywords:** *Smen*, microbiota, lactic acid bacteria, yeasts, metabarcoding, volatile organic compounds

## Abstract

In Algeria, *Smen* is a fermented butter produced in households using empirical methods. *Smen* fermentation is driven by autochthonous microorganisms; it improves butter shelf-life and yields highly fragrant products used as ingredients in traditional dishes as well as in traditional medicine. The present study is aimed at investigating microbial diversity and dynamics during Algerian *Smen* fermentation using both culture-dependent and culture-independent approaches, as well as by monitoring volatile organic compound production. To reach this goal, fifteen *Smen* samples (final products) produced in households from different regions in Algeria were collected and analyzed. In addition, microbial and volatile compound dynamics at the different stages of *Smen* manufacturing were investigated for one *Smen* preparation. The results showed that *Smen* is a microbiologically safe product, as all hygiene and safety criteria were respected. The dominant microorganisms identified by both techniques were LAB and yeasts. *Lactococcus* spp. and *Streptococcus thermophilus* were the main bacterial species involved in spontaneous raw milk fermentation preceding butter-making, while lactobacilli and enterococci were the only bacteria found to be viable during *Smen* maturation. Regarding fungal diversity, yeast species were only recovered from two mature *Smen* samples by culturing, while different species (e.g., *Geotrichum candidum*, *Moniliella* sp.) were identified in all samples by the culture-independent approach. Using microbial analysis of a single batch, many of these were found viable during manufacturing. Concerning the volatile profiles, they were highly diverse and characterized by a high prevalence of short chain fatty acids, methylketones, and esters. Correlation analysis between microbial diversity and volatile profiles showed that several yeast (*Moniliella* sp., *K. marxianus*) and LAB (e.g., *Lactococcus* spp., *S. thermophilus)* species were strongly correlated with one or more volatile organic compound families, including several ethyl esters and methyl ketones that can be linked to pleasant, sweetly floral, fruity, buttery, and creamy odors. This study clearly identified key microorganisms involved in *Smen* fermentation and maturation that could be used in the future for better fermentation control and improvement of quality attributes.

## 1. Introduction

Fermented foods constitute one of the most important diet components in many communities around the world, and represent about one-third of diets worldwide [1,2]. Traditional fermentation was empirically developed centuries ago by ancient human communities, and was used together with smoking, drying, and salting processes to preserve foods for human consumption as well as to provide new organoleptic or nutritional qualities to the final products [3,4]. This constituted a crucial step in human food culture history [1]. Fermented food products harbour complex microbial communities composed of bacteria, yeast, and/or filamentous fungi. These may be naturally present or deliberately added as starter or secondary cultures. This microbiota, with their continuously evolving and successive microbial community structures, play a central role in product-manufacturing [5]. The subtleties of the character of fermented foods as well as product shelf-life and safety are largely determined by microbiota composition, evolution, and functions [6].

Many fermented foods and beverages are produced worldwide; however, many of them have not yet been subjected to scientific study, and the biological and microbiological bases of their fermentation processes are poorly understood [7]. Understanding their microbial diversity could lead to the identification of quality biomarkers, and this knowledge could be exploited to design specific starter cultures to produce safer and higher quality foods [8].

Africa is a very large reservoir of fermented foods derived from a wide variety of raw materials including crops, cereals, oil seeds, roots, and milk [8,9]. These fermentation methods are often carried out at small scales and in households without the use of starters, and are therefore naturally driven. Among African countries, North African countries share many traditional foods which have been passed from one generation to another [10], such as *Smen*. *Smen* is a traditional fermented butter made from raw whole milk (cow, goat, or ewe’s milk either alone or as a mixture) by traditional methods [11]. Produced in Mahgreb countries, it is defined as a rancid butter obtained from raw salted butter (8% to 10%) made from spontaneously acidified milk (*Raib*) and matured in the dark under anaerobic cool conditions (13 to 15 °C) for a period of 1 to 12 months, and sometimes for even longer time periods [10,11]. This fermented butter is highly aromatic, and is especially used to improve the taste of many traditional dishes, in particular meat and pastries [11,12]. Products that are matured for long time-periods are used in traditional medicine for treating ailments such as coughs [11]. It is worth noting that the name (*Smen* or *Dhan* in Algeria, *Smen* in Morocco, *Semna* in Egypt, and *Samin* in Sudan) and preparation method can differ from one country to another.

**Figure 1:**
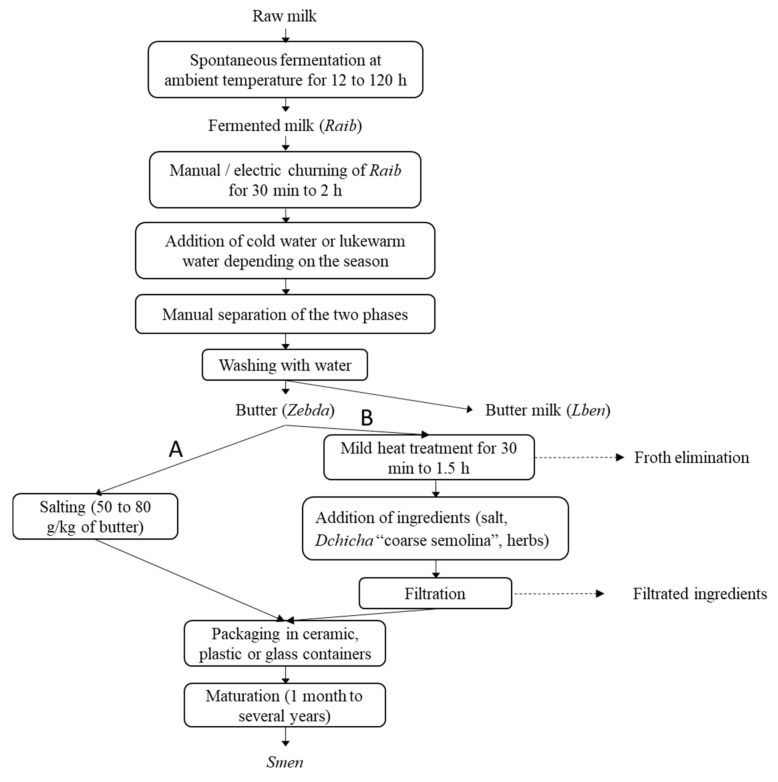
Flow diagram showing the traditional *Smen* production process according to the salting (**A**) and heat-treatment (**B**) methods.

In Algeria, *Smen* preparation methods vary from one region to another, and two main preparation modes are used (Figure 1). The first method consists of butter salting with a salt concentration between 50 and 80 g/kg of butter, and the second method consists of heat treatment of the butter followed by addition of one or more ingredients, including salt, coarse semolina, and herbs. After packing in a traditional ceramic container or other containers, a maturation step is applied, with a duration varying from one month to several years [11].

This fermented butter has been the subject of many scientific studies [12,13,14,15,16,17]. However, no detailed studies have ever characterized its microbial diversity, including both bacteria and fungi, using culture-dependent and culture-independent methods. Indeed, in the studies cited above, description of microbial diversity was achieved using culture-dependent methods and focused mainly on lactic acid bacteria and in a few cases yeast diversity. It is well established that culture-dependent methods are time-consuming, although they can reveal a large number of species in many ecosystems, including food-associated ones. In addition, only the cultivable microbiota are obtained with the selected media; it should be noted that certain microorganisms may be in a viable but non-cultivable state, and thus go undetected [18]. On the other hand, culture-independent methods are readily used to monitor evolving microbial populations over space or time and to understand microbial biodiversity in an ecosystem without using culture media [5]. It is worth mentioning that culture-dependent methods remain crucial for understanding the molecular adaptations of microbial guilds, especially those with potential biotechnological applications [19]. Moreover, use of combined approaches is of clear interest to fully understand and preserve microbiological diversity within a given ecosystem. In this context, the aim of the present study was to investigate for the first time the microbial diversity and dynamics involved in Algerian *Smen* fermentation by applying both culture-dependent and culture-independent approaches, as well as to identify the volatile organic compounds produced. To reach this goal, fifteen *Smen* samples (final product) produced in households were collected from different Algerian regions, while *Smen* microbial and volatile compound dynamics were investigated for one household preparation.

## 2. Materials and Methods

### 2.1. Sampling

#### 2.1.1. Collected *Smen* Samples

A total of fifteen samples were collected from households in different regions of Algeria (Batna, Biskra, El Oued, Jijel, Khenchela, Setif and Ouargla). The collected samples were prepared according to traditional methods, as previously described [11]; all information gathered on these products, including maturation age and milk type, is provided in Table 1. Samples were transported and stored at 4 °C for culture-dependent microbial analyses, and portions of each sample were stored at −20 °C for further analysis, including culture-independent microbial analysis.

#### 2.1.2. Dynamic Follow-Up of a *Smen* Preparation

In order to follow the microbial diversity and dynamics at different *Smen* preparation stages, *Smen* was prepared in a household in the region of Setif using locally produced butter from fresh raw milk in a small dairy. To prepare butter, 30 L of raw cow’s milk was spontaneously fermented at ambient temperature for 48 h. After milk coagulation, the coagulum (called *Raib*) was churned with a traditional churn for 55 min. During churning, warm milk was added to the mixture in order to favor agglomeration of butter grains. At the end of churning, butter was recovered using a perforated ladle; the butter yield was about 750 g. Then, at the household, butter was washed several times to remove buttermilk and impurity traces. The washed butter was salted with two tablespoons (approximately 6%) of salt, well homogenized, and covered for one week to remove water, then packaged in a glass recipient and left at room temperature for a further maturation period of six months.

Different samples were collected during manufacture, i.e., raw milk, *Raib*, added milk during churning, butter, added salt, and *Smen* after one, two, three, and six months of maturation. In addition, churn and container surfaces were sampled using a wet swabbing technique. To do this, a sterile cotton swab was moistened by immersion in 10 mL of saline solution (0.9% NaCl) in a 20 mL sterile container and the swab was rubbed on the sampling site. All samples were transported at 4 °C and analyzed directly in the laboratory using culture-dependent microbiological analyses. Milk, *Raib* and swab suspensions were centrifugated at 4000 g for 10 min and the cell pellet was stored at −20 °C for further analysis. For the other samples, an aliquot was directly stored at −20 °C for further analysis.

### 2.2. Microbiological Analyses

#### 2.2.1. Safety and Hygienic Quality

Microbiological analyses of butter and *Smen* samples were performed on the aqueous phase as previously described [20], with minor modifications. Briefly, each sample was fragmented using a sterile knife and 2.5 g was placed in a tube with 2.1 mL of 1/4 Ringer’s solution (2.25 g/L NaCl, 0.105 g/L KCl, 0.12 g/L CaCl_2_, 0.2 g/L NaHCO_3_). After vigorous homogenization, the mixture was incubated at 40 °C until fusion, and separation of the two phases was then carried out by centrifugation for 10 min at 3500 rpm. The aqueous phase was recovered with a pipette and used for serial dilutions. Milk and *Raib* samples were serially diluted in 1/4 Ringer’s solution, and inoculated on appropriate media.

Concerning hygienic quality assessment, total aerobic mesophilic bacteria were enumerated on plate count agar (Condalab, Madrid, Spain) after incubation at 30 °C for 72 h [21], while total and thermotolerant coliforms were enumerated using violet red bile lactose agar (Condalab, Spain) after incubation at 37 and 44 °C for 48 h, respectively [22]. Sulfite reducing anaerobic bacteria were enumerated on meat liver agar (Condalab, Madrid, Spain) after incubation at 37 °C for 48 h [23].

Coagulase positive staphylococci were enumerated on Baird Parker agar (Condalab, Madrid, Spain) after incubation at 37 °C for 24 h followed by confirmation of typical colonies of *Staphylococcus aureus* after microscopic characterization, Gram staining, catalase, and DNase production tests [24]. *Salmonella* detection was performed as follows: briefly, 25 g of each sample was homogenized with 225 mL of buffered peptone water (Condalab, Madrid, Spain) and incubated at 37 °C for 18 h followed by an enrichment in Müller–Kauffmann tetrathionate and selenite cystine broths at 37 °C and 45 °C for 24 h. Enrichment cultures were then plated on Hektoen agar and brilliant green bile lactose agar (Condalab, Madrid, Spain) and incubated at 37 °C for 24 h [25].

#### 2.2.2. Enumeration and Isolation of Lactic Acid Bacteria and Yeasts

Lactic acid bacteria (LAB) were enumerated on MRS agar (Condalab, Madrid, Spain) and incubated anaerobically at 30 °C for 72 h, while yeast and molds were enumerated on Sabouraud chloramphenicol agar (Condalab, Madrid, Spain) incubated at 25 °C for 3 to 7 days. LAB and yeast colonies were randomly picked from agar plates containing between 30 and 300 colonies, then purified by successively restreaking them twice using the same medium. In addition, presumptive LAB isolates were subjected to microscopic observation, Gram staining, and catalase tests. Overall, a total of 121 lactic acid bacteria and 102 yeast isolates were obtained from the studied samples. Isolates were stored at −20 °C in MRS for lactic acid bacteria and BHI broth containing 30% (*v*/*v*) glycerol for yeast prior to molecular identifications.

#### 2.2.3. Identification of Lactic Acid Bacteria and Yeast Isolates

M13 RAPD-PCR fingerprinting was first used for isolate dereplication based on their respective band patterns. Freshly grown colonies scraped from MRS or Sabouraud dextrose agar and incubated as described above, were suspended in 200 μL sterile water and stored at −20 °C. Total DNA was extracted using the FastDNA Spin kit following manufacturer’s instructions (MP Biomedicals, Illkirch-Graffenstaden, France). RAPD-PCR fingerprinting was performed using M13 primer (5′-GAGGGTGGCGGCTCT-3′) [26]. DNA amplification was carried out in an Analytik Jena thermal cycler using the following conditions: 95 °C for 5 min; 45 cycle;: 95 °C for 1 min, 36 °C for 1 min, and a final extension at 72 °C for 4 min. PCR sample aliquots (10 μL) were analyzed using 1.2% (*w*/*v*) agarose gels (Promega, Charbonnières-les-Bains, France) in 1X TAE buffer at 120 V for 1.33 h and then visualized with Midori Green staining (Nippon Genetics, Düren, Germany). RAPD band patterns were analyzed using the BioNumerics software (version 6.6; Applied Maths) and 1–3 representative isolates (82 LAB and 48 yeast isolates) of each band pattern were selected for sequencing.

Selected LAB isolates were identified by 16S rRNA gene sequencing [27] using the primers 16S-F (5′-CCGAATTCGTCGACAACAGAGTTTGATCCTGGCTCAG-3′) and 16S-R (5′-CCCGGGATCCAAGCTTACGGCTACCTTGTTACGACTT-3′) while yeast identification was performed by sequencing of the D1/D2 region of the 26S rRNA gene [28] with NL1 (5′-GCATATCAATAAGCGGAGGAAAAG-3′) and NL4 (5′-GGTCCGTGTTTCAAGACGG-3′) primers. PCR products were verified by agarose gel electrophoresis (0.8% *m*/*v* in 1X TAE buffer), and were sequenced by Eurofins sequencing platform (Eurofins Genomics, Ebersberg, Germany) using the same primers. The sequences were assembled into contigs using DNA baser software and the sequences were compared with the GenBank database using the “Basic Local Alignment Search Tool” (BLAST) (https://www.ncbi.nlm.nih.gov/BLAST, accessed on 1 December 2021) for assignment to the closest known relatives.

### 2.3. Metataxonomic Analysis of Bacterial and Fungal Communities

#### 2.3.1. Sample Preparation and DNA Extraction

Prior to DNA extraction from *Smen* or butter samples, a 20-g aliquot was solubilized with 90 mL of 2% trisodium citrate previously heated to 42 °C, then the mixture was homogenized for 3 min in a Stomacher Bag^®^ (Interscience, Saint Nom la Bretêche, France). Aliquots of 50 mL were centrifuged at 8420× *g* for 15 min at 4 °C and the supernatant and fat layer were removed. For milk and *Raib* samples, pellets stored at −20 °C and obtained as described above were solubilized in 10 mL of 2% trisodium citrate previously heated at 42 °C and vortexed vigorously, and the mixture was then centrifuged at 8420× *g* for 15 min at 4 °C. Pellets from swab samples stored at −20 °C were processed directly. 

Total DNA extraction was performed using the DNeasy^®^ Blood and Tissue Kit (Qiagen, Hilden, Germany) with an additional enzymatic lysis step as described by Penland et al. [29]. Briefly, after thawing pellets at room temperature, they were resuspended in 400 µL of lysis buffer (Tris-HCl 20 mM at pH 8.0, EDTA 2 mM, 1% Triton X-100 supplemented with 20 mg/mL lysozyme and 50 U mutanolysin). Rnase A (10 µL from a 1 mg/mL stock solution) and lyticase (10 µL at 200 U) were added separately to each sample. Samples were then incubated at 37 °C for 2 h and the mixture was gently shaken at regular time-intervals. After enzymatic treatment, a mechanical lysis step was performed after addition of 300 mg of zirconium beads using a bead mill (MM400, Retsch GmbH, Haan, Germany) set to a speed of 30 Hz for 1 min. A final treatment with proteinase K (20 mg/mL) was applied for 1 h at 54 °C. Remaining extraction and purification steps were performed according to the manufacturer’s instructions. DNA quantification was performed with Nanodrop spectrophotometer and DNA was stored at −20 °C.

#### 2.3.2. PCR and Metagenetic Sequencing Conditions

PCR was carried out using the primers S-D-bact-0341-b-S-17 (5′-CCTACGGGNGGCWGCAG-3′) and S-D-BAct-0785-a-A-21 (5′-GACTACHVGGGTATCTAATCC-3′) to amplify the V3–V4 regions of the bacterial 16S RNA [30]. For fungi, ITS3f (5′-GCATCGATGAAGAACGCAGC-3′) and ITS4_Kyo1 (5′-TCCTCCGCTTWTTGWTWTGC-3′) primers were used to amplify the ITS2 region [31].

Amplifications and sequencing steps were successively performed in the same run at the Genome Quebec sequencing platform (McGill University, Montreal, QC, Canada) using Illumina Miseq PE300 230 technology to generate 2 × 300 bp reads. The sequences obtained in this study were submitted to DDBJ\EMBL\GenBank under the accession numbers provided in Tables S6 and S7.

#### 2.3.3. Bioinformatic and Phylogenetic Analysis

Sequence data were analyzed using the FROGS pipelines (Find Rapidly OTU with Galaxy Solution) with the Galaxy platform (https://galaxy.migale.inra.fr/, accessed on 26 March 2020). Paired-end reads were first merged using Flash, then reads were clustered into OTUs using the SWARM algorithm, with an aggregation distance of 3. Chimera removal was performed using the UCHIME method on the VSEARCH tool. Prior to taxonomic affiliation, an abundance filter was applied to screen sequence clusters; sequences with relative abundance below 5 × 10^−5^ were excluded. Finally, assignment was performed using the 16S SILVA Pintail100–138 and ITS UNITE Fungi 8.2 databases for 16S and ITS data, respectively [32]. Multi-affiliations were corrected using the NCBI website. OTU affiliated with chloroplast and mitochondrial sequences were removed from the 16S dataset.

#### 2.3.4. Biodiversity and Statistical Analysis

Alpha and beta diversity analyses were performed using the QIIME 2 pipeline [33] and the Calypso software tool v8.84 [34] after normalization to the lowest sequence number in a sample and total sum normalization of count data combined with square root transformation (Hellinger transformation). Principal Coordinate Analysis (PCoA) based on Bray–Curtis distances was performed to compare biodiversity distribution at the OTU level between different categories (milk origin, geographical region, preparation method). Statistical differences between groups were evaluated using an Adonis test. Furthermore, the linear discriminant analysis (LDA) effect size (LEfSe) algorithm was applied to identify the representative bacterial and fungal taxa of each fermentation stage as well as of each collected sample.

### 2.4. Physicochemical Analysis

pH was measured using a pH meter. For *Smen* and butter samples, 10 g was melted and thoroughly mixed, then the electrode was directly placed into the sample. Milk and *Raib* pH were measured by inserting the electrode into 10 mL samples. These measurements were performed in triplicate. Moisture content was determined by heating 5 g of each *Smen* sample in a 103 °C oven until a constant weight was reached. For acid index, 2.5 g of *Smen* sample was dissolved in a mixture of diethyl ether/ethanol (1:1, *v*/*v*) previously neutralized with a KOH solution (0.1 N). The mixture was then titrated with KOH solution (0.5 N) in the presence of phenolphthalein as a pH indicator [35].

Determination of salt content was performed as follows: 5 g of each sample was solubilized in 100 mL of boiling water and titrated with silver nitrate solution at 55 °C in the presence of potassium chromate as an indicator [36]. Titratable acidity for milk and *Raib* was determined using the AOAC method; 25 mL of each sample was titrated with a solution of NaOH (0.11 N) in the presence of phenolphthalein as an indicator [37].

Volatile compounds were analyzed by headspace (HS) gas chromatography-mass spectrometry (GC-MS) using a Turbomatrix HS-40 trap, Clarus 680 gas chromatograph, and Clarus 600 T quadrupole mass spectrometer (PerkinElmer, Courtaboeuf, France). Samples of 2.5 ± 0.05 g were placed in 20 mL vials (Perkin Elmer vial) and stored at −20 °C until analysis. Compounds were eluted on an Elite WAX ETR column (30 m × 0.25 mm × 0.25 µm; Perkin Elmer, Waltham, MA, USA), with helium as the mobile phase, in the following conditions: initial temperature 35 °C maintained for 10 min, then increased at 5 °C/min up to 230 °C. The MS was operated within a mass range of *m*/*z* 29–206 and detection was by ionization impact at 70 eV [38].

Volatile compounds were identified by comparison to retention indexes, mass spectral data of standards, and from the NIST 2008 Mass Spectral Library data (Scientific Instrument Services, Ringoes, NJ, USA). GC-MS data were processed as described by Pogačić et al. [38]. Volatile compounds were quantified using the abundance of one selected mass fragment (m/z) in arbitrary units.

Differences between volatile profiles for each sample were investigated by comparing the abundance of identified compounds using principal component analysis under R software. Hierarchical clustering of the identified compounds was performed in order to investigate their abundance and dynamics in each sample using Ward’s minimum variance linkage and the Euclidian distance method with R software.

### 2.5. Correlation Analyses between Microbial Diversity and Volatile Profiles

In order to define the relationship between microbial diversity and aroma production, a Spearman rank correlation test was performed between the culture-independent and volatile compound data; *p* values were adjusted using Benjamini–Hochberg correction and significance level was set at 0.05 [39]. Results for species showing at least one significant correlation were visualized on a heat-map with hierarchical clustering using Ward’s minimum variance linkage and the Euclidean distance method. Correlations with *p* > 0.05 were considered null and set to r = 0. All analyses were performed with R software using the FactoMiner, Factoextra, Hmisc, Psych, and ggplots packages [40,41]. 

## 3. Results 

### 3.1. Microbial Diversity and Biochemical Profiles of Smen Samples Produced in Algerian Households

#### 3.1.1. Safety and Hygienic Quality

The fifteen *Smen* samples satisfied all tested safety and hygienic quality criteria as coagulase-positive staphylococci counts were below 2 log_10_ CFU/g, *Salmonella* spp. were absent, and total and thermotolerant coliforms as well as sulfite-reducing anaerobic bacteria were below their respective detection thresholds (Table 2). Total aerobic mesophilic bacteria were only detected in seven samples. All samples except O1 were produced using the salting method; counts varied between 3.2 and 5.4 log_10_ CFU/g. Lactic acid bacteria were enumerated for the same seven samples, with counts reaching up to 6.7 log_10_ CFU/g. Among these samples, two were characterized by the presence of yeasts, with counts up to 4.7 log_10_ CFU/g.

#### 3.1.2. Lactic Acid Bacteria and Yeast Diversity Using Culture-Dependent Analysis

A total of 76 LAB and 25 yeast isolates were obtained from seven and two *Smen* samples, respectively. Following M13 RAPD-PCR dereplication, 52 LAB and nine yeast isolates representing the different band patterns and considered different strains, were identified to the species level by sequencing either the 16S rRNA or D1-D2 domain of the 26S rRNA genes, respectively.

**Figure 2:**
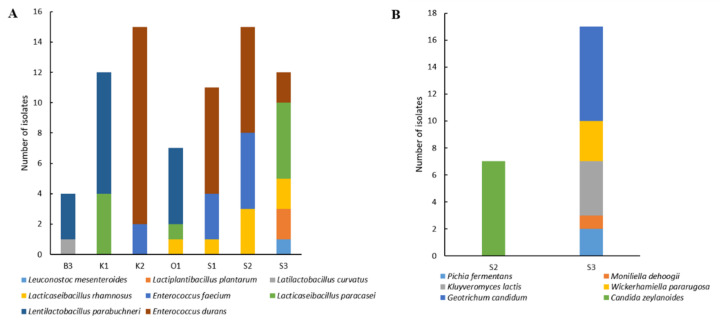
Bacterial (**A**) and fungal (**B**) diversity in *Smen* samples collected from Algerian households determined by culture-dependent analyses.

As shown in Figure 2A, among LAB, enterococci and lactobacilli represented 51.31% and 47.36%, respectively, of the total isolates, and were thus clearly dominant. For enterococci, two species were identified, *Enterococcus faecium* and *Enterococcus durans*, which were dominant in three samples, S1, S2, and K2. Lactobacilli diversity was much higher and they dominated in the four remaining samples with, in decreasing order of frequency, *Lentilactobacillus parabuchneri*, *Lacticaseibacillus paracasei*, *Lacticaseibacillus rhamnosus*, *Lactiplantibacillus plantarum*, and *Latilactobacillus curvatus.* One *Leuconostoc mesenteroides* isolate was identified in one sample.

Concerning yeast diversity (Figure 2B), six species were identified. Sample S2 contained only one species, *Candida zeylanoides*, while five species were identified in the S3 sample. *Geotrichum candidum* was the most dominant, followed by *Wickerhamiella pararugosa, Kluyveromyces lactis*, *Pichia fermentans*, and *Moniliella dehoogii.*

#### 3.1.3. Microbial Diversity Using Metagenetic Analyses

##### Bacterial Diversity 

After total DNA extraction from the studied *Smen* samples, nine samples (B4, B5, K1, K2, O1, S1, S2, S3, S5) yielded sufficient DNA for metagenetic analysis. With the exception of O1, all samples were prepared using the salting method, and six (K1, K2, O1, S1, S2, S3) had bacterial populations detectable by culturing. From these nine *Smen* samples, a total of 297,203 quality filtered sequences were generated, which were clustered into 160 OTUs. After normalization to the lowest read number in a sample (17,291), the number of observed species (richness) ranged between 44 and 110, while the Chao1 index (estimated richness) ranged from 45 to 97.5, evenness from 0.15 to 0.69, the Shannon index from 0.56 to 3.14, and Simpson’s index of diversity from 0.18 to 0.90 (Table S1). Significant differences within bacterial communities were found taking into account categorical variables that were correctly represented among the studied samples. Indeed, the Chao1 index was significantly higher in *Smen* samples made from cow’s milk than in *Smen* obtained from goat’s milk (*p* ˂ 0.05). In addition, the Chao1 and Simpson indices were significantly different (*p* < 0.05) according to the region of origin, with those collected from Setif having the highest values for both indices. It is worth mentioning that all *Smen* samples from the Setif region were made out of cow’s milk. Finally, sample O1 (prepared using a heat-treatment method) harboured the lowest species richness, Chao1, and Simpson index. 

**Figure 3:**
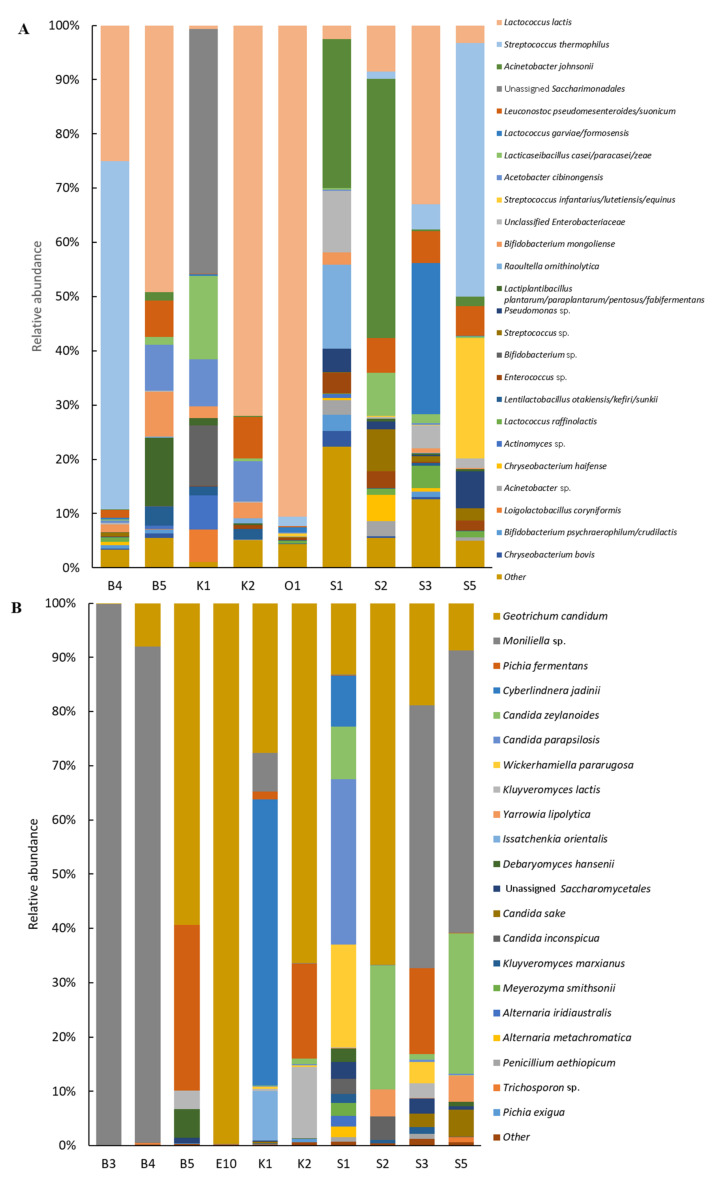
Bacterial (**A**) and fungal (**B**) diversity in *Smen* samples from different Algerian households determined by a metagenetic approach.

Compositional analysis of *Smen* samples revealed that OTUs belonged to eight phyla, with *Bacillota* representing 67.5% of total sequences followed by *Pseudomonadota* (20.4%), *Actinomycetota* (5.7%), Candidate Phyla Radiation (CPR)/”Patescibacteria” (5%), and *Bacteroidota* (1.4%). As shown in Figure 3A, which represents the top 25 species distribution in all samples, *Smen* samples harboured complex bacterial communities. Overall, *Lactococcus lactis*, *Streptococcus thermophilus*, and *Acinetobacter johnsonii* were the main representative species. *L. lactis* was dominant in the B4, B5, K2, S3, and O1 samples, with a relative abundance of 25.0%, 49.2%, 72.0%, 33.0%, and 90.6%, respectively, although it was observed in smaller proportions in K1 (0.7%), S2 (8.5%), S1 (2.5%), and S5 (3.3%). Other *Lactococcus* species included *Lactococcus raffinolactis* and *Lactococcus garvieae*/*formosensis*, the latter having a high relative abundance in the S3 sample. *S. thermophilus* was present in five samples, B4, S3, S2, O1, and S5, and was dominant in B4 (64.2%) and S5 (46.7%). Another *Streptococcus* sp. related to *Streptococcus infantarius*/*lutetiensis*/*equinus* was identified in sample S5. *A. johnsonii* dominated in S1 and S2, with a relative abundance of 27.5 and 47.7%, respectively, while an unidentified member of the *Saccharimonadales* order belonging to the Candidate Phyla Radiation (CPR)/”Patescibacteria” was dominant in the K1 sample. In contrast to the culture-dependent results, lactobacilli and enterococci were not the most dominant taxa, although reads from several *Enterococcus* and *Lactobacillus* related-species were found to be part of the top 25 taxa present in these *Smen* samples. Other species encountered in the different samples, with mean relative abundances over 1% of total sequences, included *Bifidobacterium*, *Acetobacter*, an unassigned member of the *Enterobacteriaceae* family, *Pseudomonas*, *Raoultella*, *Enterococcus*, and *Chryseobacterium* spp.

Beta-diversity among *Smen* bacterial communities was investigated using PCoA based on Bray–Curtis distances; variance analysis of these distances was then performed using a multivariate permutation Adonis test. At the OTU level, milk origin was the only factor which significantly shaped bacterial communities (*p* < 0.05, R^2^ = 0.257). It should be noted that the effect of *Smen* preparation methods on bacterial community structuring could not be evaluated as only one sample prepared according to that method was included in our dataset. 

To further identify bacterial taxa which were over- or underrepresented in *Smen* produced from cow or goat milk, a linear discriminant analysis (LDA) coupled with effect size measurements (LEfSe) was performed at the genus, species, and OTU levels. Six genera, twelve species, and seventeen OTUs exhibited significantly different abundances depending on milk origin. Indeed, the microbiota of goat milk *Smen* were enriched in OTUs related to *Acinetobacter cibinongensis*, *Lentilactobacillus otakiensis/kefiri/sunkii*, and *Levilactobacillus brevis*, while those from cow’s milk were enriched in OTUs from species of the genera *Streptococcus*, *Chryseobacterium*, *Pseudomonas*, *Enterococcus*, *Serratia*, *Acinetobacter*, *Kocuria*, and *Brachybacterium*. 

##### Fungal Diversity 

The ITS2 region metabarcoding analysis generated a total of 646,883 quality-filtered sequences from ten samples (B3, B4, B5, E10, K1, K2, S1, S2, S3, S5) clustered into seventy OTUs. After rarefaction to the lowest number of sequences in a sample (50,092), richness ranged from 21 to 53, while the Chao1 index ranged from 19 to 54, evenness from 0.007 to 0.56, the Shannon index from 0.02 to 2.16 and Simpson’s index of diversity from 0.004 to 0.83 (Table S2). Comparison of alpha-diversity indices according to categorical variables showed that the Shannon and Simpson indices were significantly different according to geographical origin, as samples collected from Setif and Khenchela presented the highest values. Sample E10, which was prepared using the heat-treatment method, harboured the lowest alpha-diversity indices.

Fungal communities from *Smen* samples were dominated by *Ascomycota* and *Basidiomycota* members, which represented 69.9 and 30.1% of total sequences, respectively. Figure 3B shows fungal species distribution in the different *Smen* samples. Overall, *Smen* mycobiota of each sample was dominated by one to five species. Interestingly, samples such as S1 and S3 were characterized by much higher diversity than the other samples, with respectively twelve and nine species (relative abundances over 1%). *G. candidum* was the only species found at high relative abundance (ranging from 8% to 99.7%) in all samples with the exception of sample B3, followed by *Moniliella* sp. (abundances over 1% in five out of ten samples). This species was the most dominant in samples where *G. candidum* relative abundance was low. Other abundant species included *P. fermentans*, *C. zeylanoides*, *K. lactis*, *Cyberlindnera jadinii*, and *W. pararugosa.*

Concerning beta-diversity analysis, as previously observed for bacterial communities, milk origin was the only factor which significantly shaped *Smen* mycobiota (*p* < 0.05, R^2^ = 0.258). LefSe analysis at the OTU level showed that *Smen* prepared from goat milk were enriched in *Pichia exigua* and *K. lactis* OTUs, whereas an OTU related to *Y. lipolytica* was more prevalent in *Smen* made from cow’s milk.

#### 3.1.4. Physico-Chemical Analysis and Volatile Profiles

The physico-chemical properties of the studied *Smen* samples are shown in Table 2. The pH values ranged between 5.2 and 3.1, and were generally below pH 4, higher pH values being found in <3 months old *Smen* samples. Moisture values did not exceed 19.9% and acid index values ranged between 2.2 and 153.7 mg KOH/g, with increased values in samples with increased maturation times. This was especially true for the B1 sample, which was matured for ten years. Salt content was variable and found between 7.4 and 0.1%, with the lowest values in two samples (i.e., Jn and O1) prepared using the heat-treatment method.

Volatile compound analyses by HS-GC-MS identified 81 compounds belonging to different families, i.e., esters (20), ketones (20), aldehydes (13), fatty acids (12), alcohols (7), furans (3), terpenes (3), styrene (1), and others (2) (Table S3 and Figure S1). 

**Figure 4:**
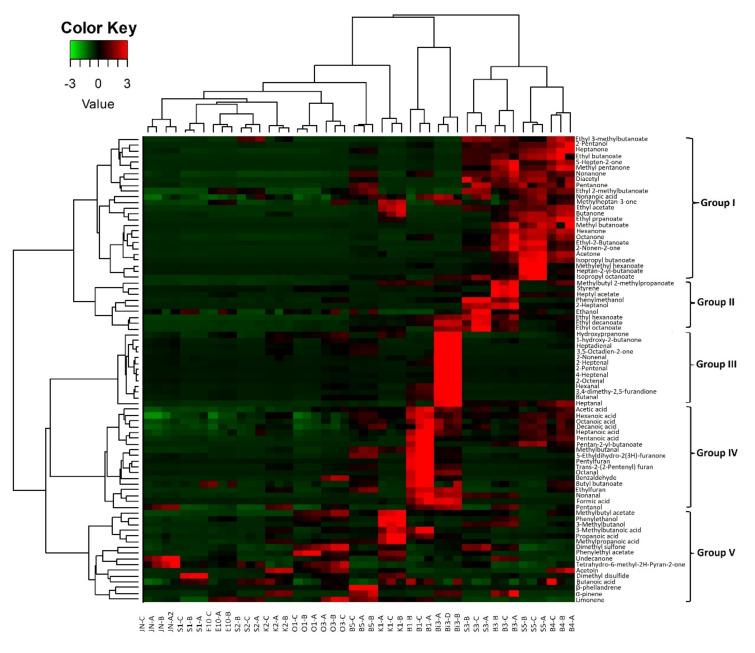
Normalized heat-map representation of volatile abundances in fifteen different *Smen* samples collected from Algerian households. Hierarchical clustering was performed using Ward’s Linkage and Euclidean distance.

Based on the abundance distribution of volatile compounds for each sample, five different groups could be differentiated (Figure 4). A first group gathered 25 compounds, mainly ketones (twelve compounds, e.g., pentanone, diacetyl, hexanone) and esters (eleven compounds, e.g., ethyl butanoate, ethyl acetate, methyl butanoate). One alcohol (2-pentanol) and one fatty acid (nonanoic acid) was identified in this group. These compounds were abundant in the B3, B4, and S5 samples, while only twelve of them were present at high abundances in the S3 sample. Group II gathered nine compounds. Among them, three compounds were present at high abundances in sample B3, i.e., methylbutyl-2-methylpropanoate, styrene, and heptyl acetate. The remaining compounds corresponded to alcohols (*n* = 3) and esters (*n* = 3) and were abundant in both B3 and S3 samples. Group III gathered thirteen compounds present at high abundances in the Bi3 sample. These compounds included nine aldehydes (e.g., heptadienal and 2-nonenal) and four ketones (e.g., hydroxypropanone, and 3,5-octadien-2-one). Group IV gathered eighteen compounds which were much more abundant in the B1 sample. These mainly corresponded to fatty acids (*n* = 7, e.g., acetic acid, hexanoic acid, octanoic acid) and aldehydes (*n* = 4; e.g., benzaldehyde and nonanal). The same fatty acids, except for pentanoic acid, were abundant in the B3 sample, whereas ethylfuran, nonanal, formic acid, and pentanol were found at high abundances in both the B1 and B3 samples. Group V gathered sixteen compounds belonging to different families, i.e., ketones, esters, alcohols, fatty acids, terpenes, and others. In this group, the K1 sample was associated with methylbutyl acetate, phenylethanol, 3-methylbutanol, 3-methylbutanoic acid, propanoic acid, methylpropanoic acid, phenylethyl acetate, dimethylsulfone, and undecanone. Acetoin was related to the K2, B4 and O3 samples. In addition, the O3 sample was associated with dimethyl sulfone, phenylethyl acetate, undecanone, and tetrahydro-6-methyl-2H-pyran-2-one, while the O1 sample was associated with dimethyl sulfone and phenylethyl acetate and Jn was associated with two ketones, undecanone and tetrahydro-6-methyl-2H-pyran-2-one.

Terpenes were found at high abundances in the B5 sample, i.e., β-phellandrene, α-pinene, limonene. Limonene was found at high abundance in the O3 sample and at lower levels in E10 and S2.

### 3.2. Microbial Dynamics and Diversity and Biochemical Profiles throughout Different Preparation Stages of Smen

#### 3.2.1. Safety and Hygienic Quality 

**Figure 5:**
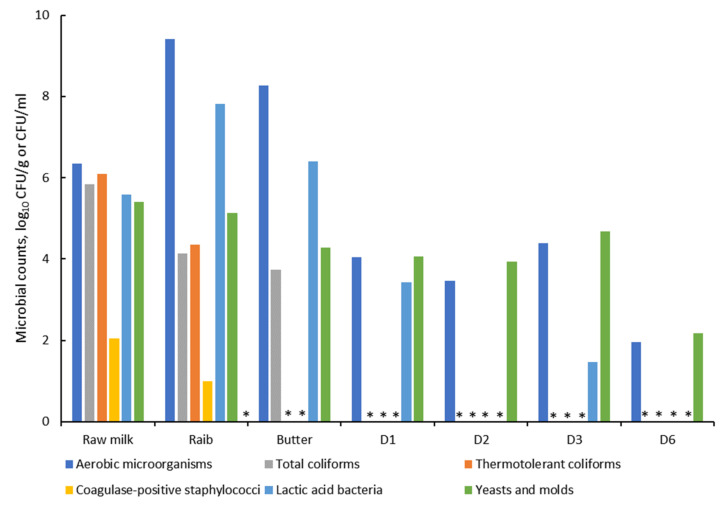
Microbial counts during the different preparation stages of *Smen* (D1, D2, D3, D6: *Smen* after one, two, three, and six months of maturation, respectively). * indicates counts below the detection threshold.

In the second part of this study, the microbial diversity and dynamics during *Smen* manufacture were investigated using culture-dependent and culture-independent approaches. As shown in Figure 5, there were important variations in microbial counts at the different *Smen* production stages. Concerning total aerobic microorganisms and LAB, their counts increased during raw milk fermentation to *Raib*, with counts reaching 9.42 and 7.81 log_10_ CFU/g, respectively. A slight decrease in the latter populations was observed in butter, followed by a gradual decrease during *Smen* maturation. It should be underlined that LAB were found at very low populations, i.e., 1.41 log_10_ CFU/g, in *Smen* matured for three months (D3), and were below the detection limit in *Smen* matured for two (D2) and six (D6) months. In contrast, yeast and mold counts remained stable during *Smen* manufacture up to three months of maturation, with counts ranging from 4 to 5 log_10_ CFU/g. For total and thermotolerant coliforms, counts were high in raw milk (~6 log_10_ CFU/mL), decreased in *Raib*, and were below detection thresholds in *Smen* matured for one month (D1) and butter for total coliforms and thermotolerant coliforms, respectively. Coagulase-positive staphylococci followed the same trend, with an initial 2 log_10_ CFU/mL population in raw milk, and were not detected after butter making. Noteworthily, *Salmonella* spp. and sulfite-reducing anaerobic bacteria were systematically absent during all *Smen* manufacturing steps.

#### 3.2.2. Lactic Acid Bacteria and Yeast Diversity Using Culture-Dependent Analysis

A total of 45 LAB and 77 yeast isolates were obtained from different preparation stages (raw milk, *Raib*, butter and *Smen*/*Dhan* with different maturation times, i.e., one and three months) of which 30 LAB and 39 yeast isolates representative of the different RAPD patterns were identified by sequencing. 

**Figure 6:**
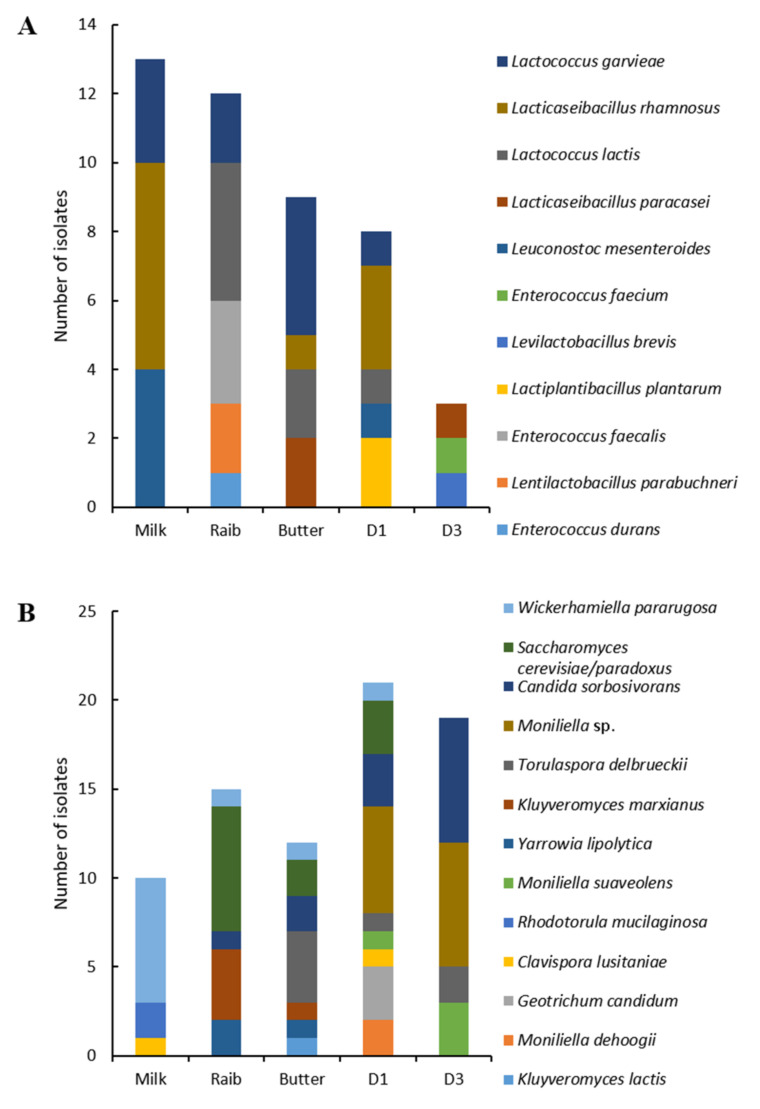
Bacterial (**A**) and fungal (**B**) diversity during the different preparation stages of *Smen* (D1, D3: *Smen* after one and three months of maturation, respectively) determined by the culture-dependent approach.

Concerning LAB diversity, eleven species including enterococci, lactobacilli, leuconostoc, and lactococci were identified, i.e., *E. faecium*, *E. durans*, *Enterococcus faecalis*, *L. plantarum*, *L. rhamnosus*, *Levilactobacillus brevis*, *L. paracasei*, *L. parabuchneri*, *L. mesenteroides*, *L. lactis*, and *L. garvieae*. Shifts in LAB communities were observed throughout the different *Smen* preparation stages (Figure 6A). Indeed, while *L. rhamnosus*, *L. mesenteroides*, and *L. garvieae* dominated in raw milk, *L. lactis*, *L. garvieae*, *L. parabuchneri*, *E. faecalis*, and *E. durans* were dominant in *Raib* and *L. paracasei*, *L. lactis*, *L. rhamnosus* and *L. garvieae* were dominant in butter. The latter three species were found in one-month matured *Smen* along with *L. mesenteroides*, previously isolated from raw milk. Only three isolates were identified in three-month matured *Smen*, and belonged to the *L. brevis*, *E. faecium*, and *L. paracasei* species.

As shown in Figure 6B, yeast isolates collected throughout *Smen* manufacturing and maturation were assigned to thirteen different species, *Clavispora lusitaniae*, *Rhodotorula mucilaginosa*, *W. pararugosa*, *Kluyveromyces marxianus*, *Saccharomyces cerevisiae*/*paradoxus*, *Y. lipolytica*, *Candida sorbosivorans*, *Torulaspora delbrueckii*, *K. lactis*, *G. candidum*, *M. dehoogii*, *Moniliella suaveolens*, and an unidentified *Moniliella* species. Noteworthily, the latter isolates which were dominant in one-month and three-month *Smen* samples likely correspond to a novel species, as their D1-D2 domain 26S rRNA gene sequences shared less than 95% similarity with *M. dehoogii*, the closest phylogenetic relative (data not shown). As previously observed for LAB, there were shifts in fungal communities during *Smen* manufacture and maturation. In raw milk, three dominant species were identified, *W. pararugosa*, *R. mucilaginosa*, *C. lusitaniae*, while in *Raib*, *R. mucilaginosa* and *C. lusitaniae* were no longer detected and instead the dominants yeasts were *S. cerevisiae* followed by *K. marxianus*, *Y. lipolytica*, *C. sorbosivorans*, and *W. pararugosa.* The butter mycobiota were quite similar to that of *Raib* except that *T. delbrueckii* was detected in addition to the previously mentioned species. After one month of maturation, *Smen* harboured species previously found in raw milk, *Raib* and butter as well as other species that were not previously detected, i.e., *G. candidum* and *Moniliella* spp. Finally, *Smen* samples matured for three months were characterized by a high dominance of *Moniliella* spp. and *C. sorbosivorans*, while other species, e.g., *S. cerevisiae* and *G. candidum*, were no longer detected.

#### 3.2.3. Microbial Diversity Using Culture-Independent Analysis

##### Bacterial Diversity Using V3–V4 Metabarcoding Analysis

Metabarcoding analysis was performed at the different stages of *Smen* preparation. In addition, this analysis was applied on potent microbial reservoirs including added milk during churning, traditional churn surface, added salt and, *Smen* containers. The total number of filtered high-quality reads generated from all samples was 368,039; these were clustered into 104 OTUs. After rarefaction to the lowest sequence number in a sample (22,483), richness ranged between 53 to 75, while the Chao1 index ranged from 54 to 79, evenness from 0.12 to 0.64, the Shannon index from 0.67 to 3.95, and Simpson’s index of diversity from 0.14 to 0.88 (Table S4). Considering only alpha-diversity indices of samples collected throughout *Smen* production and maturation, the raw milk bacterial microbiota were characterized by the highest species richness (75), Simpson index (0.88), evenness (0.64), and Shannon index (3.95). While species richness remained quite high after milk fermentation, with values between 53 (*Raib*) and 72 (butter), the Simpson, evenness and Shannon indexes of samples collected after milk fermentation, namely, *Raib*, butter and *Smen* after one, three, and six months maturation, decreased to values ranging from 0.14 to 0.24, 0.12 to 0.18, 0.67 and 1.1, respectively. This observation indicates that following raw milk fermentation, the bacterial microbiota of the latter samples were unequally distributed and less diverse, i.e., dominated by a relatively limited number of species. 

**Figure 7:**
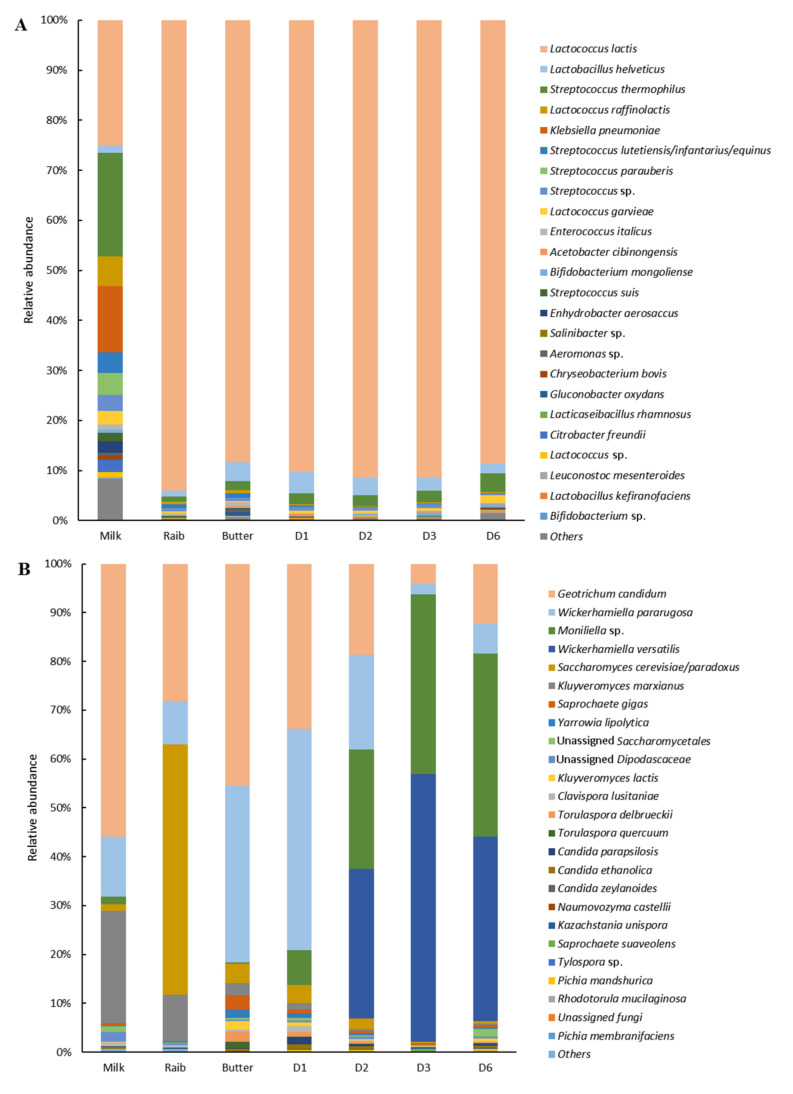
Bacterial (**A**) and fungal (**B**) diversity during *Smen* preparation stages (D1, D2, D3, D6: *Smen* after one, two, three, and six months of maturation, respectively) determined by a metagenetic approach.

This result was confirmed by the compositional analysis of samples throughout *Smen* manufacturing (Figure 7A). Indeed, raw milk harboured a complex bacterial community, with fourteen species present at a relative abundance >1%. These species corresponded on the one hand to members of the *Bacillota* phylum, including lactococci (*L. lactis*, *L. raffinolactis*, *L. garvieae*), streptococci (*S. thermophilus*, *Streptococcus lutetiensis*/*infantarius*/*equinus*, *Streptococcus parauberis*, *Streptococcus suis*), and to a lesser extent to enterococci (*Enterococcus italicus*) and lactobacilli (*Lactobacillus helveticus*). On the other hand, raw milk was characterized by the presence of members of the *Pseudomonadota* phylum either belonging to the *Enterobacterales* (i.e., *Klebsiella pneumoniae*, *Citrobacter freundii*) and *Pseudomonadales* order (i.e., *Enhydrobacter aerosaccus*). In contrast, the bacterial diversity of *Raib*, butter, and *Smen* samples during maturation was dominated by few species, with, in decreasing order of abundance, *L. lactis* (with relative abundances ranging from 88% to 91%), *L. helveticus*, and *S. thermophilus*. Other minor species (relative abundance < 1%) included those observed in raw milk, e.g., enterococci, other lactococci (i.e., *L. garvieae* and *L. raffinolactis*), lactobacilli species (i.e., *Lactobacillus kefiranofaciens*) and *Leuconostoc* spp. (i.e., *L. mesenteroides*) as well as members of the *Pseudomonadota* phylum. Moreover, while bacterial species distribution in *Smen* containers was, as expected, quite similar to that of butter, *Smen* species distribution in milk added during churning, at the traditional churn surface, and in the added salt were quite different (Figure S2). In particular, the milk added during churning and the surface of the traditional churn were characterized by high relative abundances of *L. helveticus* and *L. raffinolactis* OTUs, respectively, whereas the salt bacterial community was dominated by OTUs related to *Haloparvum* and *Salinibacter* sp.

##### Fungal Diversity Using ITS2 Metabarcoding Analysis

After filtering out low-quality and chimeric sequences, a total of 368,039 high-quality sequences were obtained and clustered into 78 OTUs. After rarefaction to the lowest number of sequences in a sample (39,443), the number of observed species (richness) ranged from 47 to 60, while the Chao1 index ranged from 49 to 90, evenness from 0.34 to 0.53, the Shannon index from 1.9 to 3.39, and Simpson’s index of diversity from 0.64 to 0.82 (Table S5). Overall, comparison of alpha-indices from the different studied samples did not show any important variations, the only exception being the salt used for butter salting, which was characterized by a high Chao1 index (i.e., 90) compared to the other samples.

Regarding fungal diversity during *Smen* manufacturing steps, our results highlighted major shifts in the dominant fungi (Figure 7B). Indeed, while the raw milk mycobiota were dominated by *G. candidum*, *K. marxianus* and *W. pararugosa*, *Raib* was dominated by the latter three species as well as *S. cerevisiae*/*paradoxus*, which was present in raw milk at low relative abundance. In butter, both *G. candidum* and *W. pararugosa* remained dominant while other species, including the other previously mentioned species as well as *C. lusitaniae*, *Saprochaete gigas*, *K. lactis*, *T. delbrueckii*, *Torulaspora quercuum*, and *Y. lipolytica*, were found at relative abundances over 1%. After one month maturation, the *Smen* mycobiota were quite similar to the one observed for butter, except that a *Moniliella* sp. was found at 7.2% relative abundance. After two, three, and six months maturation, the latter species became progressively dominant together with *Wickerhamiella versatilis*, while the relative abundance of *G. candidum* and *W. pararugosa* decreased. LefSe analysis at the OTU level confirmed that *Smen* samples were enriched with *Moniliella* sp. and *W. versatilis*-related OTUs. Interestingly, regarding potent fungal reservoirs encountered during *Smen* manufacturing (Figure S2), most harbored a high relative abundance of *G. candidum* (with the exception of the *Smen* containers) and *W. pararugosa*. In addition, the churning equipment and added milk during churning harboured high relative abundances of *S. gigas* and *K. marxianus*, while the salt and *Smen* containers appeared to be important sources of *Moniliella* sp. and to a lesser extent of *W. versatilis*.

#### 3.2.4. Physico-Chemical Analysis and Volatile Profiles

Physico-chemical changes during *Smen* preparation are shown in Table 3. The pH value of raw milk was 6.7 and, after 48 h fermentation, *Raib* pH was 4.2 and associated with an increase in Dornic acidity from 16.3 to 47. After churning, butter pH slightly increased to 4.5, then, after *Smen* preparation and one month maturation, decreased by ~0.5 units to reach 4 and remained constant after six months maturation. The acid index value for butter was 6.7 mg KOH/g. It increased during *Smen* maturation and ranged between 10.1 and 21.1 mg KOH/g after one and six months, respectively. Moisture values slightly decreased before and after *Smen* preparation. They varied between 22.6% for butter and between 15.9% and 14.8% after one and six months’ maturation, respectively. Prior to salting, butter contained 0.2% while after salting and one month maturation, it reached 1% before slowly increasing to 1.3% at the end of maturation.

During *Smen* maturation, a large number of volatile compounds were generated (Figure S3). A total of 81 compounds were identified, as described above in Section 3.1.4. Sample projection using PCA (Figure S4) showed that the first two dimensions, which accounted for 44.10% and 21.02%, respectively, separated samples according to their maturation time (0 maturation time (butter) on the left to 180 days (D6) maturation on the right). As shown in Figure S4, the matured samples (D3, D6) were found in the right quadrant, while the other samples with 30 (D1) and 60 (D2) maturation days were identified in the upper quadrant. Butter samples formed a separate group in the lower left quadrant. The D3 and D6 samples were associated with many compounds, mainly esters (*n* = 16, e.g., methylbutyl acetate, ethyl acetate, methyl butanoate), ketones (*n* = 15, e.g., diacetyl, octanone, acetone) as well as other compounds such as alcohols (*n* = 2, e.g., 2 pentanol, pentanol), aldehydes (*n* = 5, e.g., butanal, benzaldehyde, heptanal), fatty acids (*n* = 6, e.g., heptanoic, hexanoic, decanoic acids), furans (*n* = 2, i.e., ethylfuran, pentylfuran), and terpenes (*n* = 3, i.e., α-pinene, β-phellandrene, limonene), while the D1 and D2 samples were specifically associated with certain alcohols (methylbutanol, phenylethanol, ethanol), aldehydes (heptadienal, 2 nonenal), esters (phenylethyl acetate, ethyl-3-methyl butanoate), fatty acids (formic acid), and ketones (dimethyl-2,5- furandione, hydroxypropanone, acetoin, dimethyl sulfone). On the other hand, the butter sample was linked to heptanal, methylbutanal, ethyl 2-methylbutanoate, and acetic acid.

**Figure 8:**
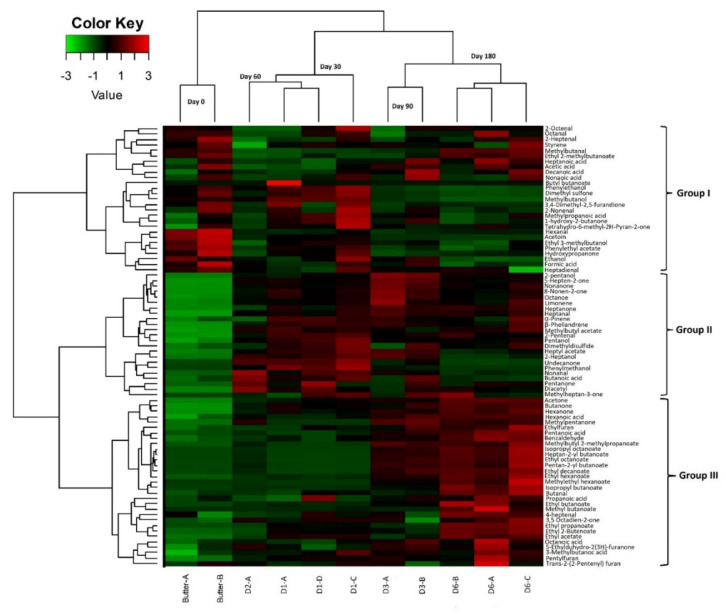
Normalized heat-map representation of volatile abundances during *Smen* maturation. Hierarchical clustering was performed using Ward’s Linkage and Euclidean distance. (D1, D2, D3, D6: *Smen* after one, two, three, and six months of maturation, respectively).

In order to investigate volatile compound dynamics during maturation, a heatmap representation was performed presenting the 81 volatile compounds by hierarchical clustering analysis (Figure 8). We were able to define three different clusters. The first group included 27 compounds: seven aldehydes (e.g., 2 octenal, 2 heptenal, hexanal), six fatty acids (e.g., heptanoic acid, decanoic acid, acetic acid), and five ketones (e.g., hydroxypropanone, acetoin, 1- hydroxy-2-butanone) together with four esters, three alcohols, one sulfone, and styrene. Certain aldehydes (2 octenal, octanal, 2 heptenal, hexanal, heptadienal) were found to be more abundant in raw butter, and their abundance gradually decreased between the first and third month of maturation. However, their abundance increased (except for hexanal) at six months maturation along with an increase in branched esters, free fatty acids, and styrene (methylbutanal, ethyl 2- methylbutanoate, butyl butanoate, heptanoic acid, acetic acid, nonanoic acid,). A slight decrease in acetoin, hydroxypropanone, phenylethyl acetate, ethanol, and formic acid was noted at the end of maturation compared to raw butter. The first month of maturation (D1) was characterized by the abundance of several compounds, mainly ketones (*n* = 3), esters (*n* = 2), aldehydes (*n* = 3), and alcohols (*n* = 3). Group II gathered 23 compounds, mainly ketones, i.e., 5-hepten-2-one, nonanone, 8-nonen-2-one, and octanone, the abundance of which increased from the first month of *Smen* maturation and continued to slightly increase to the end of maturation. The same trend was observed for 2-pentanol, limonene, heptanal, α-pinene, β-phellandrene, and methylbutyl acetate. At the mid-maturation stage (30 and 60 days), several compounds, i.e., pentanone, diacetyl, methylheptan-3-one, pentanol, 2-heptanol, nonanal, 2-heptanal, butanoic acid, heptyl acetate, phenylmethanol, undecanone, and dimethyl disulfide, presented their highest abundance before slightly decreasing at the end of maturation (90 days and 180 days). Finally, group III gathered 31 compounds including fourteen esters (e.g., isopropyl octanoate, ethyl octanoate, methylethyl hexanoate), six ketones (e.g., acetone, butanone, hexanone), five fatty acids (e.g., hexanoic acid, pentanoic acid, propanoic acid), three aldehydes (benzaldehyde, butanal, 4- heptenal), and three furans (ethylfuran, pentylfuran, trans-2,2-pentyl furan). The abundance of these compounds was the highest at the end of maturation.

#### 3.2.5. Correlation between Volatile Profiles and Microbiota during Maturation

**Figure 9:**
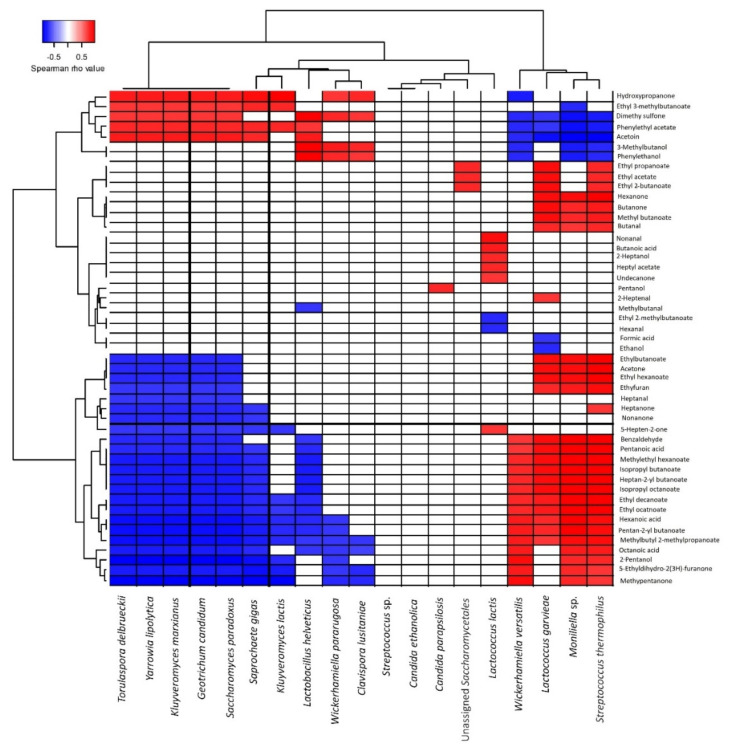
Spearman correlation matrices between microbial species composition and volatile compounds during *Smen* maturation (butter and *Smen* samples matured for one to six months). Only significant correlations are shown (*p* < 0.05). Positive correlations are indicated in red while negative ones are indicated in blue.

To better understand the nature of the correlations between volatile compounds and microbial species, Spearman’s correlations were calculated between the two parameters, i.e., volatile profiles and independent microbial data for which a relative abundance cut-off was set to ˃0.5%. Different profiles could be differentiated (Figure 9). Nine species presented strong negative correlations (r ˃ 0.7) with esters i.e., *L. helveticus* (*n* = 9), *G. candidum* (*n* = 12), *S. cerevisiae/paradoxus* (*n* = 12), *K. marxianus* (*n* = 10), *S. gigas* (*n* = 9), *Y. lipolytica* (*n* = 11), *C. lusitaniae* (*n* = 4), *K. lactis* (*n* = 4), and *T. delbrueckii* (*n* = 10), while opposite correlations were observed for *L. garvieae* (*n* = 14), *S. thermophilus* (*n* = 11), *W. versatilis* (*n* = 8), and *Moniliella* sp. (*n* = 12). On the other hand, *Y. lipolytica* and *T. delbrueckii* showed a high positive correlation with phenylethyl acetate (r ˃ 0.8). By comparison, three species showed the highest correlations with different esters (r ˃ 0.9): *Moniliella* sp. with ten esters and *L. garvieae* and *S. thermophilus* with eight different esters.

*Moniliella* sp. and *W. versatilis* were the only species that showed strong positive correlations (r > 0.8) with fatty acids, i.e., octanoic acid, pentanoic acid, and hexanoic acid, while other species showed strong negative correlations with the same fatty acids. Regarding acetoin production, it was related to *L. helveticus*, *G. candidum*, *K. marxianus*, *S. gigas*, *Y. lipolytica* and *T. delbrueckii*. Several species showed strong positive correlation with certain ketones, i.e., *Moniliella* sp. and *K. marxianus*. The production of 2-pentanol was related to *S. thermophilus* (r = 0.96), *Moniliella* sp. (r = 0.8), and *W. versatilis* (r = 0.88). On the other hand, for dimethyl sulfone, positive correlations were noted for *L. helveticus*, *K. marxianus*, *Y. lipolytica*, *C. lusitaniae*, and *T. delbrueckii.*

## 4. Discussion

In the present study, the microbial diversity and dynamics as well as physico-chemical and volatile profiles of Algerian *Smen*, a spontaneously fermented butter produced using empirical methods, were investigated using several microbial, molecular and biochemical methods. To the best of our knowledge, this is the first time such an extensive study has been carried out on this fermented product. 

Regarding *Smen* safety and hygiene, the present study confirmed, in agreement with a previous study [13], that *Smen* is a safe product, as all tested hygiene and safety criteria were satisfactory. This result is not surprising, as several hurdles during the *Smen* manufacturing process are encountered and contribute to its safety and salubrity. Indeed, spontaneous fermentation of raw milk to *Raib* by LAB leads to competition-exclusion phenomena as well as production of antimicrobial compounds (e.g., lactic acid) and a pH decrease. D Products are also rich in free fatty acids, the latter showing an increase during the maturation process. In the present study, the impact of these hurdles on microbial counts, and in particular on coagulase-positive staphylococci and total and thermotolerant coliforms, was clearly noticeable throughout the different *Smen* preparation stages. Indeed, coagulase-positive staphylococci, thermotolerant coliforms, and total coliforms were all below the detection threshold in butter and one-month matured *Smen*, respectively. It is worth noting that, among the fifteen products collected from Algerian households, all were satisfactory regarding hygiene and safety criteria and only two samples harboured viable yeasts with counts of ~4 log_10_ CFU/g, while seven samples harboured viable bacteria with counts quite similar to LAB, mainly identified as enterococci and lactobacilli. While lactobacilli are generally recognized as safe, enterococci, especially *E. faecium*, have a paradoxical status, as they are responsible for nosocomial infections and can transfer antibiotic resistance genes and can play a positive role in many fermented foods as well, including dairy products [42]. In this regard, it would be of interest to investigate *Smen* enterococci isolates for virulence genes and their acquired antibiotic resistance properties, as well as to go further in the taxonomical identification of *E. faecium* isolates, in order to determine whether they belong to clade A (hospital associated) or B (community associated), the latter clade being recently reassigned to *Enteroccocus lactis* [43]. 

Regarding *Smen* microbial diversity, two separate complementary studies were undertaken using both culture-dependent and metagenetic approaches. First, the microbial diversity and volatile profiles of *Smen* samples collected from different households were investigated. As expected, there were important differences in the results obtained by each microbiological approach, as culture-dependent approaches revealed certain lactobacilli and enterococci as well as yeast species, in agreement with previous studies [44,45,46,47,48] in samples matured for up to one year, with counts up to ~6 and ~4 log_10_ CFU/g, respectively. In contrast, metagenetic analysis, because it is based on total DNA either derived from live and dead cells, provided a general overview of microbial species that left their DNA signature during *Smen* manufacture and maturation. In *Smen* samples (those where sufficient DNA quantity was extracted for downstream metagenetic analysis), bacterial communities were found to be quite diverse, with *L. lactis*, *S. thermophilus*, and/or *A. johnsonii*, being the main representative species. *L. lactis* and *S. thermophilus* were previously identified as the main species involved in the spontaneous fermentation of raw milk to *Raib*, called *Rayeb* in Morocco, which can be consumed as is, processed further to a traditional white cheese called *Jben*, or churned to obtain buttermilk (Moroccan and Algerian lben, Tunisian leben, Egyptian, Iraqi and Lebanese laban) and raw butter (*Zebda*), the latter being used for *Smen* production [16,17,49,50,51,52]. Concerning *A. johnsonii*, identified at a high relative abundance in two samples (S1 and S2), together with other Gram-negative species belonging to the *Pseudomonadota* phylum and enterococci, their higher relative abundances, when compared to LAB species, is likely the result of either a fermentation deviation during spontaneous milk fermentation or the use of spoiled raw milk, and/or of poor hygiene quality, as this species is considered a major milk spoilage species [53]. 

For the first time, the present study provided an overview of *Smen* mycobiota, which have been systematically overlooked to date. While most identified species (e.g., *G. candidum*, *C. zeylanoides, K. lactis*) are regularly encountered in raw milk and dairy products [54,55,56,57], an interesting finding was the high prevalence of *Moniliella* spp., a black yeast from the *Basidiomycota* phylum. Members of this genus are generally considered as food spoilage agents in acidic and/or low a_w_ foods, including fat-rich dairy products [58,59]. Its presence in *Smen* is not surprising, as this genus comprises species which are considered acid-resistant, xerophilic, and fermentative; as demonstrated in the second part of this study, *Moniliella* sp. can grow during *Smen* maturation. Interestingly, the type species of *Moniliella spathulata* (CBS 241.79) was originally isolated from Indian *Ghee* made from buffalo milk [60]. 

The investigation of the volatile profiles of *Smen* collected from households led to the identification of 81 compounds belonging to different families, mainly derived from fat metabolism; most of them were similar to those previously detected in Moroccan *Smen* [12,61]. Indeed, as previously reported [12], in addition to short chain (C_4_ to C_10_) fatty acids, which are important contributors to *Smen* aromatic profiles, key compounds included methylketones and esters, particularly butanoic and hexanoic ethyl esters and 2-pentanone. Enterococci (e.g., *E. faecalis* and *E. durans*) [62], lactobacilli (e.g., *L. paracasei, L. plantarum, L. rhamnosus*) [63,64], and yeast (e.g., *C. zeylanoides, G. candidum, M. dehoogii, W. pararugosa*) [47,65,66] species isolated in the present study have been previously shown to possess lipase and esterase activities. Thus, through lipolysis and conversion of free fatty acids to other compounds (e.g., short-chain fatty acids, methylketones, and ethyl esters), they likely contribute to *Smen* maturation during storage, as further discussed below. Moreover, as for Moroccan *Smen* [12], these products displayed diverse volatile profiles with no clear clustering according to maturation time, milk origin, or preparation method. This result is not surprising given the fact that *Smen* manufacturing processes can vary from one household to another and these processes rely on the activity of complex microbial communities composed of autochthonous microorganisms.

Several hypotheses driven by the aforementioned results were confirmed in the second part of this study by following microbial community and volatile profile dynamics during different stages of *Smen* manufacture and maturation. It was found that *L. lactis* dominated immediately after raw milk fermentation, as confirmed by both the culture-dependent and culture-independent analyses. It then became progressively subdominant after churning and subsequent maturation, as shown by the culture-dependent approach, while cultivable bacteria in matured *Smen* belonged to lactobacilli and enterococci. This result clearly highlighted that the strong dominance of *L. lactis*, revealed using a metagenetic approach, in *Smen* collected from households could be the result of a bias due to the presence of VBNC, or more likely, dead cells, as *L. lactis* can be isolated on MRS medium; a similar observation concerning discrepancies between culture-dependent and metagenetic results regarding *L. lactis* prevalence in dairy products was reported for Pélardon cheese [29]. To overcome this potential bias, the use of a DNA stain (e.g., propidium monoazide) which can bind to DNA from dead cells, thus rendering it inaccessible for PCR amplification, could have been useful. There was another discrepancy between the results of the culture-dependent and culture-independent approaches regarding *G. candidum* prevalence; while this species was found to dominate in *Raib* by metagenetic analysis, it was not isolated. We have no clear explanation for this result. 

The results of this part of this study defined the possible technological/functional roles played by bacterial and fungal species encountered during *Smen* manufacturing and maturation. In the studied batch, *L. lactis* and to a lesser extent *S. thermophilus* and *L. helveticus* were the main bacterial species involved in raw milk spontaneous fermentation to *Raib*. During this process, lactose is fermented into lactate, while citrate can be metabolized by wild *L. lactis* to CO_2_, acetic acid, and α-acetolactate. The latter can be enzymatically transformed into acetoin or chemically degraded to diacetyl, which has buttery and creamy notes [61]. In raw butter, acetate and acetoin were found at high abundances, while diacetyl was present at higher abundances in *Smen* matured for one month. During maturation, *Smen* was progressively enriched in short-chain fatty acids, including even-numbered (e.g., butanoic, hexanoic, octanoic and decanoic acids) and odd-numbered (e.g., pentanoic, heptanoic, nonanoic) fatty acids. These compounds are characterized by strong odors described as fatty, rancid buttery, ripened cheese, whey-flowery, cheesy-musty, rancid buttery, and soapy [61]. The increase in fatty acid abundance can be attributed to residual milk lipase activity as well as to the lipase and esterase activities of LAB and more importantly yeasts. In this regard, strong positive correlations were found between fatty acids and *W. versatilis* and *Moniliella* sp. In addition to short-chain fatty acids, ethyl esters, i.e., ethyl acetate, ethyl butanoate, and ethyl hexanoate, were the most dominant identified compounds in *Smen*, especially at the end of maturation. Ethyl esters, which have pleasant, sweetly floral, and fruity odors [61], can be formed via two different reactions catalyzed by microbial esterases/lipases, namely, esterification and alcoholysis. This explains the decrease in abundances of alcohols such as ethanol, methylbutanol, and phenylethanol at the early stages of maturation and the increase in ester abundances at the end. Strong positive correlations were noted between ethyl esters and *L. helveticus*, *L. garvieae*, *S. thermophilus*, *Moniliella* sp. and *W. versatilis*, all of which are already known for their esterase activities [67,68,69,70]. The second-most abundant compounds were ketones. Their abundance increased after the first month of maturation. Ketones are produced through oxidation of fatty acids to β-ketoacids and decarboxylation [71]. These compounds, which have fruity, floral, and musty notes, were strongly correlated with *K. marxianus* and *Moniliella* sp. Nevertheless, to the best of our knowledge, *K. marxianus* is not a ketone producer [72]. *Moniliella* spp. are able to produce γ-decalactone and other γ-lactones and δ-lactones [73], however, nothing is known regarding their ability to produce methyl ketones. In contrast, *G. candidum*, which was identified at high relative abundance using metagenetics, is a very well-known ketone producer [65].

## 5. Conclusions

In conclusion, the present study provided new knowledge about the microbial diversity, dynamics, and volatile profiles of Algerian *Smen* and identified the key microorganisms during *Smen* manufacturing and maturation as well as their relation with volatile compound production. The diversity and abundances of the volatile compounds identified here were highly related to maturation time and lipolysis degree as well as to their autochthonous microbiota. This knowledge and the collection of microorganisms built through this study could be useful to select LAB and yeast strains to design future specific starter and non-starter cultures for *Raib* and *Smen* production at a larger scale, e.g., cooperatives of local producers to better control these fermentation processes and improve *Smen* quality attributes [11].

## Figures and Tables

**Table 1:** Description of collected *Smen* samples.

Samples	Milk Origin	Maturation Time	Region	Preparation Method	Storage Temperature
B1	Cow	10 years	Batna	Salting	Ambient
B3	Cow	<1 year	Batna	Salting	Ambient
B4	Cow	<1 year	Batna	Salting	Ambient
B5	Goat	2 years	Batna	Salting	Ambient
Bi3	Ewe and goat	<1 year	Biskra	Heat-treatment	Refrigerated
E10	Goat	1 year	El Oued	Heat-treatment	Ambient
Jn	Cow	<3 months	Jijel	Heat-treatment	Ambient
K1	Goat	6 months	Khenchela	Salting	Ambient
K2	Goat	10 months	Khenchela	Salting	Ambient
O1	Goat	1 year	Ouargla	Heat-treatment	Refrigerated
O3	Goat	1 year	Ouargla	Heat-treatment	Ambient
S1	Cow	6 months	Setif	Salting	Ambient
S2	Cow	2 months	Setif	Salting	Ambient
S3	Cow	1 months	Setif	Salting	Ambient
S5	Cow	2 years	Setif	Salting	Ambient

**Table 2:** Physicochemical properties and microbial counts (log_10_ CFU/g) of the fifteen different *Smen* samples produced in Algerian households.

Samples	pH	Moisture (%)	Acid Index (mg KOH/g)	Salt Content (%)	Total Aerobic Mesophilic Bacteria	Total Coliforms	Thermo-Tolerant Coliforms	LAB ^1^	Yeast and Molds	Coagulase-Positive Staphylococci	*Salmonella* spp.	Sulphite-Reducing Anaerobic Bacteria
B1	3.1 ± 0.0	19.9	153.7 ± 1.7	ND ^1^	<1	<1	<1	<1	<2	<2	n.d. ^3^	n.d.
B3	3.5 ± 0.0	12.2	35.9 ± 0.0	ND	4.5	<1	<1	2.3	<2	<2	n.d.	n.d.
B4	3.4 ± 0.0	12	40.3 ± 0.1	ND	<1	<1	<1	<1	<2	<2	n.d.	n.d.
B5	3.6 ± 0.0	12.6	31.4 ± 0.0	ND	<1	<1	<1	<1	<2	<2	n.d.	n.d.
Bi3	3.8 ± 0.0	0.8	19.1 ± 1.1	ND	<1	<1	<1	<1	<2	<2	n.d.	n.d.
E10	ND	ND	ND	ND	<1	<1	<1	<1	<2	<2	n.d.	n.d.
Jn	4.3 ± 0.0	0	3.9 ± 0.1	0.1 ± 0.0	<1	<1	<1	<1	<2	<2	n.d.	n.d.
K1	3.3 ± 0.0	12.7	6.7 ± 0.0	3.3 ± 0.0	5.2	<1	<1	5.1	<2	<2	n.d.	n.d.
K2	3.8 ± 0.0	9.0	14.6 ± 1.5	2.2 ± 0.0	4.1	<1	<1	3.1	<2	<2	n.d.	n.d.
O1	5.2 ± 0.0	0	2.2 ± 0.0	0.1 ± 0.0	3.2	<1	<1	3.3	<2	<2	n.d.	n.d.
O3	ND	0	2.2 ± 0.0	ND	<1	<1	<1	<1	<2	<2	n.d.	n.d.
S1	3.3 ± 0.0	12.8	3.3 ± 0.0	7.4 ± 0.1	3.7	<1	<1	4.5	<2	<2	n.d.	n.d.
S2	3.6 ± 0.0	7.3	3.6 ± 0.0	3.9 ± 0.9	5.3	<1	<1	5.5	4.2	<2	n.d.	n.d.
S3	4.4 ± 0.0	10.5	4.4 ± 0.6	1.4 ± 0.0	5.4	<1	<1	6.7	4.7	<2	n.d.	n.d.
S5	ND	2.5	43.8 ± 1.9	ND	<1	<1	<1	<1	<2	<2	n.d.	n.d.

ND, Not Determined due to limited sample size; ^1^ LAB, lactic acid bacteria; n.d., not detected.

**Table 3:** Physicochemical properties of samples collected during *Smen* manufacture stages.

Sample	pH	Moisture (%)	Dornic Acidity (D°)	Salt Content (%)	Acid Index (mg KOH/g)
Milk	6.7 ± 0.0	ND	16.3 ± 0.0	ND	ND
Raib	4.2 ± 0.0	ND	47.0 ± 0.0	ND	ND
Butter	4.5 ± 0.1	22.6	ND	0.20 ± 0.0	6.7 ± 0.0
1-month *Smen* (D1)	4.0 ± 0.1	15.9	ND	1.10 ± 0.0	10.1 ± 1.1
2-month *Smen* (D2)	4.3 ± 0.1	15.1	ND	1.10 ± 0.0	18.4 ± 0.0
3-month *Smen* (D3)	4.1 ± 0.0	15.0	ND	1.20 ± 0.0	19.1 ± 1.1
6-month *Smen* (D6)	4.0 ± 0.0	14.8	ND	1.30 ± 0.1	21.1 ± 0.3

ND, Not Determined.

## Data Availability

Original Research sequence data have been deposited in the Sequence Read Archive (SRA) database at the NCBI under accession PRJNA775076.

## Supplementary Materials

The following supporting information can be downloaded at: https://www.mdpi.com/article/10.3390/microorganisms10040736/s1, Table S1: Alpha-diversity indices of bacterial communities from *Smen* samples collected in different households, Table S2: Alpha-diversity indices of fungal communities from *Smen* samples collected in different households, Table S3: Volatile compounds identified in different *Smen* samples using HS-GC-MS, Table S4: Alpha-diversity indices of bacterial communities from *Smen* samples throughout the different preparation stages of *Smen*, Table S5: Alpha-diversity indices of fungal communities from *Smen* samples throughout the different preparation stages of *Smen*, Table S6: Accession numbers of metabarcoding sequences (16S), Table S7. Accession numbers of metabarcoding sequences (ITS), Figure S1: Examples of chromatograms obtained using headspace (HS) gas chromatography-mass spectrometry (GC-MS) of *Smen* samples collected in different households, Figure S2: Bacterial (A) and fungal (B) diversity in potent microbial reservoirs encountered during *Smen* manufacturing revealed using a metagenetic approach, Figure S3: Examples of chromatograms obtained using headspace (HS) gas chromatography-mass spectrometry (GC-MS) of *Smen* during maturation, Figure S4: PCA plots of volatile compounds from the 81 volatile compounds detected throughout *Smen* maturation. PCA plot of individuals (A) and variables (B) showing principal component 1 (Dim1, 44.10%) versus 2 (Dim2, 21.02%).
