# Supplementary material for: Deciphering the Microbiota and Volatile Profiles of Algerian Smen, a Traditional Fermented Butter
Source: Microorganisms. 2022 Mar 29;10(4):736. doi: 10.3390/microorganisms10040736 (PMC9028027; doi:10.3390/microorganisms10040736)
Supplement: Supplementary file 1 [file microorganisms-10-00736-s001.zip › microorganisms-1632873-supplementary.pdf]

Supplementary Tables

Table S1. Alpha-diversity indices of bacterial communities from *Smen* samples collected in different households

SampleID	Seqs/Sample	chao1 Ave.	equitability Ave.	goods_coverage Ave.	observed_species Ave.	shannon Ave.	simpson Ave.
B4	17290	83.937	0.284	0.999	73.9	1.762	0.523
B5	17290	69.962	0.46	0.999	61.3	2.733	0.723
K1	17290	54.224	0.472	0.999	47.2	2.622	0.745
K2	17290	51.264	0.315	1	48.5	1.765	0.468
O1	17290	46.8	0.151	1	43.9	0.825	0.182
S1	17290	113.149	0.68	1	106.5	4.579	0.905
S2	17290	88.349	0.548	0.999	76.1	3.427	0.824
S3	17290	102.822	0.511	0.999	94.9	3.354	0.803
S5	17290	98.031	0.434	0.999	86.6	2.795	0.723

Table S2. Alpha-diversity indices of fungal communities from *Smen* samples collected in different households

SampleID	Seqs/Sample	chao1 Ave.	equitability Ave.	goods_coverage Ave.	observed_species Ave.	shannon Ave.	simpson Ave.
B3	45130	21	0.23	1	20.7	1.006	0.319
B4	45130	30.176	0.102	1	28.4	0.492	0.161
B5	45130	28.3	0.375	1	26.9	1.782	0.587
E10	45130	23.401	0.008	1	20.1	0.036	0.005
K1	45130	44.883	0.355	1	41.9	1.914	0.633
K2	45130	31.575	0.347	1	29.3	1.693	0.529
S1	45130	54.068	0.554	1	51.6	3.151	0.833
S2	45130	37.903	0.283	1	34.2	1.443	0.499
S3	45130	41.825	0.515	1	41.4	2.768	0.749
S5	45130	56.333	0.466	1	53	2.671	0.766

Table S3. Volatile compounds identified in different *Smen* samples using HS-GC-MS

Family	Compounds	Odor character	Formula	MW (g/mol)	Ion (m/z)	RI calcule (AT)	RI Literature
Alcohols	Ethanol	sweet	C_4H_8O	72.11	46	937	925-955
	2-Pentanol*	balsamic	$C_5H_{12}O$	88.15	45	1133	1081-1153
	3-Methylbutanol	fermented, fruity, pungent	$C_5H_{12}O$	88.148	70	1225	1180-1230
	Pentanol	fruit	$C_5H_{12}O$	88.15		1270	1241-1271
	2-Heptanol	herb	$C_7H_{16}O$	116.2	83	1335	1280-1344
	Phenylmethanol	floral, rosy, phenolic	C_7H_8O	108.14	60	1898	1821-1909
	Phenylethanol		$C_8H_{10}O$	122.16	79	1942	1859-1947
Aldehydes	Butanal	pungent, green	C_4H_8O	72.11	72	859	855-911
	Methylbutanal	malt	$C_5H_{10}O$	86.13	58	905	884-924
	Hexanal	grass, tallow, fat	$C_6H_{12}O$	100.16	56	1077	1062-1111
	2-Pentenal	almond, malt, pungent	C_5H_8O	84.12	84	1130	1043-1145
	Heptanal	fat, citrus, rancid	$C_7H_{14}O$	114.19	96	1182	1160-1196
	4-heptenal	biscuit, cream	$C_7H_{12}O$	112.17	68	1238	1216-1267
	Octanal	fat, soap, lemon, green	$C_8H_{16}O$	128.21	84	1283	1273-1306
	2-Heptenal	fat, citrus, rancid	$C_7H_{12}O$	112.17	83	1314	1287-1340
	Nonanal	fat, citrus, green	$C_9H_{18}O$	142.24	96	1389	1375-1409
	2-Octenal	green, nut, fat	$C_8H_{14}O$	126,2	70	1432	1392-1466

	Heptadienal	nut, fat	$C_7H_{10}O$	110.15	81	1514	1451-1522
	Benzaldehyde	almond, burnt sugar	C_7H_6O	106.12	105	1536	1488-1529
	2-Nonenal	orris, fat, cucumber	$C_9H_{16}O$	140.22	83	1548	1497-1582
Esters	Ethyl_Acetate	pineapple	C_4H_{82}	60.05	70	869	856-917
	Ethyl propanoate	fruit, win	$C_5H_{10}O_2$	130.18	102	944	910-977
	Methyl_butanoate	fruity, winey	$C_5H_{10}O_2$	88.105	102	989	943-1008
	Ethylbutanoate	ether, fruit, sweet	$C_6H_{12}O_2$	116.16	88	1030	1010-1071
	Isopropyl_butanoate	sweet, fruity	$C_7H_{14}O_2$	130.18	71	1035	
	Ethyl-2-methylbutanoate*	pungent, fruit	$C_7H_{14}O_2$	130.18	102	1045	1015-1073
	Ethyl-3-methylbutanoate	sweet, fruity	$C_7H_{14}O_2$	130.18	85	1063	1041-1114
	Methylbutyl_acetate	sweet, fruity	$C_7H_{14}O_2$	130.18	70	1130	1112-1143
	Ethyl-2-Butenoate*	fruity	$C_6H_{10}O_2$	114.14	99	1165	
	Pentan-2-yl butanoate		$C_9H_{18}O_2$	158.24	71	1211	1214-1260
	Butyl butanoate		$C_8H_{16}O_2$	144.21	101	1214	1183-1243
	Ethyl hexanoate		$C_8H_{16}O_2$	144.21	88	1232	1222-1258
	Methylethyl hexanoate	apple peel, fruit	$C_9H_{18}O_2$	158.24	158	1232	1223-1236
	Methylbutyl 2-methylpropanoate*		$C_9H_{18}O_2$	158.24	70	1263	1183-1234
	Heptyl acetate	flower	$C_9H_{18}O_2$	158.24	98	1372	1360-1401

	Heptan-2-yl butanoate*	fruity, winey	$C_{11}H_{22}O_2$	186.29	115	1398	1388-1401
	Isopropyl octanoate		$C_{11}H_{22}O_2$	186.29	84	1446	1419-1471
	Ethyl octanoate		$C_{10}H_{20}O_2$	172.26	88	1446	1428-1466
	Ethyl decanoate	fruity, winey	$C_{12}H_{24}O_2$	200.32	101	1636	1595-1678
	Phenylethyl acetate		$C_{10}H_{12}O_2$	164.2	104	1812	1775-1862
Fatty acids	Hexanoic acid	fruity, fatty sour	$C_6H_{12}O_2$	116.16	60	1846	1810-1860
	Butanoic acid	acidic, buttery	$C_4H_8O_2$	88.11	60	1623	1596-1653
	Acetic acid	sour	$C_2H_4O_2$	60.05	60	1475	1403-1479
	Formic acid	pungent	CH_2O_2	46.025	46	1534	1470-1533
	Heptanoic acid	rancidity	$C_7H_{14}O_2$	130.18	87	1951	1915-1997
	Octanoic acid	oily, rancid, capric	$C_8H_{16}O_2$	144.21	60	2059	2024-2067
	Nonanoic acid	fatty	$C_9H_{18}O_2$	158.24	129	2169	2128-2202
	Propanoic acid	pungent, rancid, soy	$C_4H_8O_2$	74.08	74	1555	1486-1560
	Pentanoic acid	sweat	$C_5H_{10}O_2$	60	60	1739	1685-1770
	Decanoic acid	rancid, fat	$C_{10}H_{20}O_2$	60	60	2275	2231-2316
	Methylpropanoic acid	/	$C_4H_8O_2$	73	73	1577	1547-1588
	3-methylbutanoic acid	green, phenolic	$C_5H_{10}O_2$	60	60	1670	1647-1686
	Ethylfuran	_	C_6H_8O	96.13	81	939	1219-1249

Furans	Pentylfuran	green bean, butter	$C_9H_{14}O$	138.21	81	1228	1282
	Trans-2-(2-Pentenyl) furan	_	$C_9H_{12}O$	136.19	107	1296	1005-1067
Ketones	Methylpentanone	mint	$C_8H_{16}O$	128.21	100	1011	1005-1067
	Tetrahydro-6-methyl-2H-Pyran-2-one		$C_6H_{10}O_2$	114.14	70	1786	1751-1830
	3,5-Octadien-2-one	geranium, metal	$C_8H_{12}O$	124.18	124	1573	1524-1610
	Undecanone	orange, fresh, green	$C_{11}H_{22}O$	170.29	58	1595	1570-1628
	3,4-dimethyl-2,5-furandione		$C_6H_6O_3$	126.11	126	1729	1680-1764
	Acetone		$C_4H_8O_2$	88.11	58	810	809-842
	Butanone		C_4H_8O	88.11	72	887	893-943
	Diacetyl	butter	$C_4H_6O_2$	86.09	86	976	951-999
	Pentanone	ether, fruit	$C_5H_{10}O$	86.13	71	985	845-980
	Hexanone	ether, grape	$C_6H_{12}O$	100.16	100	1075	1069-1116
	Heptanone	soap	$C_7H_{14}O$	114.19	114	1180	1178-1190
	5-Hepten-2-one	metal	$C_7H_{12}O$	112.17	112	1218	1249
	Methylheptan-3-one*		$C_8H_{16}O$	128.21		1262	
	Acetoin	butter, cream	$C_4H_8O_2$	88.11	88	1278	1265-1287
	Octanone	herb, butter, resin	$C_8H_{16}O$	128.21	128	1279	1265-1309
	Hydroxypropanone		$C_3H_6O_2$	74.08	74	1295	1266-1326
	1-Hydroxy-2-butanone		$C_4H_8O_2$	88.11	57	1373	1351-1399

	Nonanone	hot milk, soap, green	$C_9H_{18}O$	142.24	58	1384	1374-1403
	8-Nonen-2-one	baked	$C_9H_{16}O$	140.22	111	1457	1473-1494
	5-Ethyldihydro-2(3H)-uranone		$C_8H_{12}O_2$	140.18	85	1695	1661-1745
Terpenes	α-Pinene	pine, turpentine	$C_{10}H_{16}$	136.23	136	1010	1000-1077
	Limonene	lemon, orange mint, turpentine	$C_{10}H_{16}$	136.23	136	1186	
	β-Phellandrene		$C_{10}H_{16}$	94.14	79	1900	1190-1220
	Dimethyl disulfide	onion, cabbage, putrid	$C_2H_6S_2$	94.2	94	1062	1036-1094
Others	Dimethyl sulfone	sulfur, burnt	$C_2H_6O_2S$	136.23	93	1196	1895-1912
Styrene	Styrene	balsamic, gasoline	C_8H_8	104.15	93	1248	1299-1293

*Tentatively identified

Table S4. Alpha-diversity indices of bacterial communities from *Smen* samples throughout the different preparation stages of *Smen*.

Description	Seqs/Sample	chao1 Ave.	equitability Ave.	goods_coverage Ave.	observed_species Ave.	shannon Ave.	simpson Ave.
Milk	22480	75.067	0.634	1	74.7	3.945	0.879
Raib	22480	61.648	0.116	1	53.5	0.665	0.137
Churn surface	22480	75.933	0.408	1	65.3	2.459	0.629
Milk churn	22480	75.5	0.548	1	74	3.405	0.807
Butter	22480	79.598	0.177	1	71.8	1.092	0.24
Salt	22480	57.144	0.322	1	52.7	1.84	0.578
Smen container surface	22480	69.091	0.243	1	62.3	1.447	0.345
Smen 1 month	22480	61.433	0.146	1	55.7	0.849	0.2
Smen 2 months	22480	53.705	0.133	1	50.3	0.752	0.175
Smen 3 months	22480	64.558	0.139	1	56.8	0.811	0.181
Smen 6 months	22480	70.41	0.164	1	59.9	0.97	0.221

Table S5. Alpha-diversity indices of fungal communities from *Smen* samples throughout the different preparation stages of *Smen*.

Description	Seqs/Sample	chao1 Ave.	equitability Ave.	goods_coverage Ave.	observed_species Ave.	shannon Ave.	simpson Ave.
Milk	39440	60.973	0.344	1	57	2.005	0.622
Raib	39440	49.75	0.34	1	49	1.911	0.644
Churn surface	39440	54.133	0.434	1	50	2.446	0.709
Milk churn	39440	49.261	0.391	1	47	2.169	0.686
Butter	39440	53.657	0.416	1	50.8	2.36	0.681
Salt	39440	53.9	0.528	1	53.9	3.039	0.755
Smen container surface	39440	90.05	0.398	1	51	2.257	0.669
Smen 1 month	39440	57.412	0.427	1	55.7	2.478	0.701
Smen 2 months	39440	60.7	0.506	1	56.7	2.95	0.819
Smen 3 months	39440	54.453	0.367	1	52.3	2.097	0.646
Smen 6 months	39440	64.858	0.491	1	60.4	2.905	0.793

Table S6. Accession numbers of metabarcoding sequences (16S)

Sample	Type of sample	ID library	Accession number
B4	Fermented butter	lib372829	SAMN23005575
B5	Fermented butter	lib372830	SAMN23102035
K1	Fermented butter	lib372831	SAMN23102456
K2	Fermented butter	lib372832	SAMN23102457
S3	Fermented butter	lib372833	SAMN23102498
S2	Fermented butter	lib372834	SAMN23102499
S1	Fermented butter	lib372835	SAMN23131947
O1	Fermented butter	lib372828	SAMN22962632
S5	Fermented butter	lib372836	SAMN22962633
Milk	Milk	lib372837	SAMN22962648
Raib	Raib	lib372838	SAMN22962661
Bio churn	Bio churn	lib372839	SAMN22962662
Butter	Butter	lib372841	SAMN22962670
Salt	Salt	lib372842	SAMN22963470
Bio recepient	Bio recepient	lib372839	SAMN22968376
D1	Fermented butter	lib372844	SAMN22968413
D3	Fermented butter	lib372846	SAMN22968438
D6	Fermented butter	lib372847	SAMN23005371
Milk churn	Milk	lib372840	SAMN23133887
D2	Fermented butter	lib372845	SAMN22968425

Table S7. Accession numbers of metabarcoding sequences (ITS)

Sample	Type of sample	ID library	Accession number
B4	Fermented butter	lib373096	SAMN22602011
B5	Fermented butter	lib373097	SAMN22645107
K1	Fermented butter	lib373098	SAMN22645411
K2	Fermented butter	lib373099	SAMN22645454
B3	Fermented butter	lib373115	SAMN22739177
S3	Fermented butter	lib373100	SAMN22739247
E10	Fermented butter	lib373116	SAMN22741321
S2	Fermented butter	lib373101	SAMN22744425
S1	Fermented butter	lib373102	SAMN22746555
S5	Fermented butter	lib373103	SAMN22785408
Milk	Milk	lib373104	SAMN22837352
Raib	Raib	lib373105	SAMN22837353
Bio churn	Bio churn	lib373106	SAMN22837354
Butter	Butter	lib373108	SAMN22837355
Salt	Salt	lib373109	SAMN22837356
Bio recipient	Bio recepient	lib373110	SAMN22837357
D1	Fermented butter	lib373111	SAMN22837358
Milk churn	Milk churn	lib373107	SAMN22895715

Supplementary Figures.

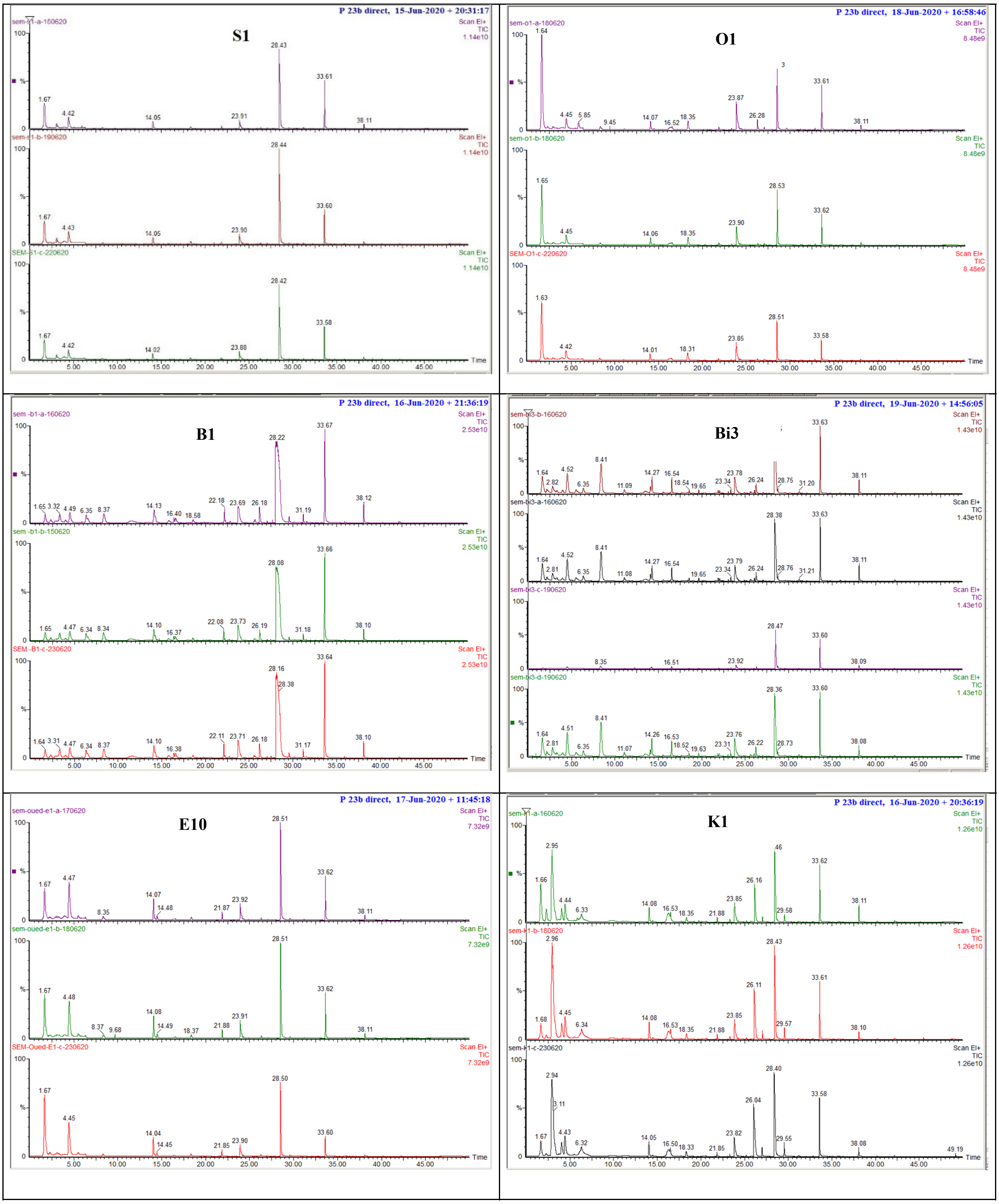

Figure S1. Examples of chromatograms obtained using headspace (HS) gas chromatography-mass spectrometry (GC-MS) of *Smen* samples collected in different households.

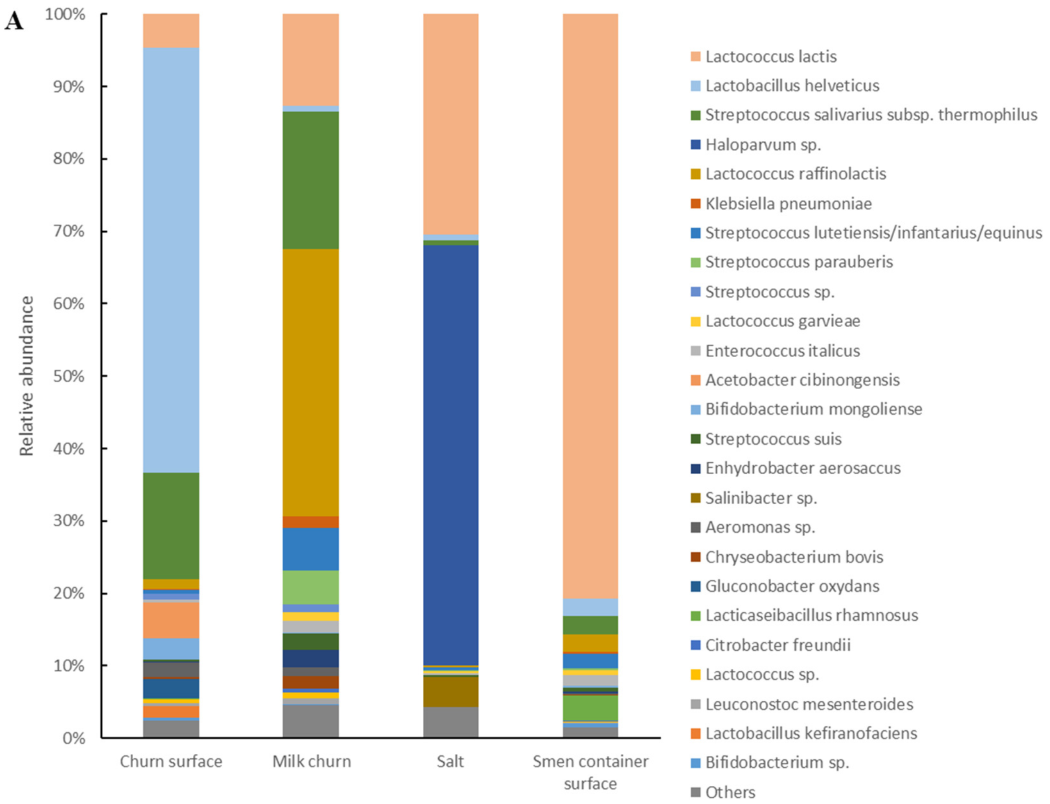

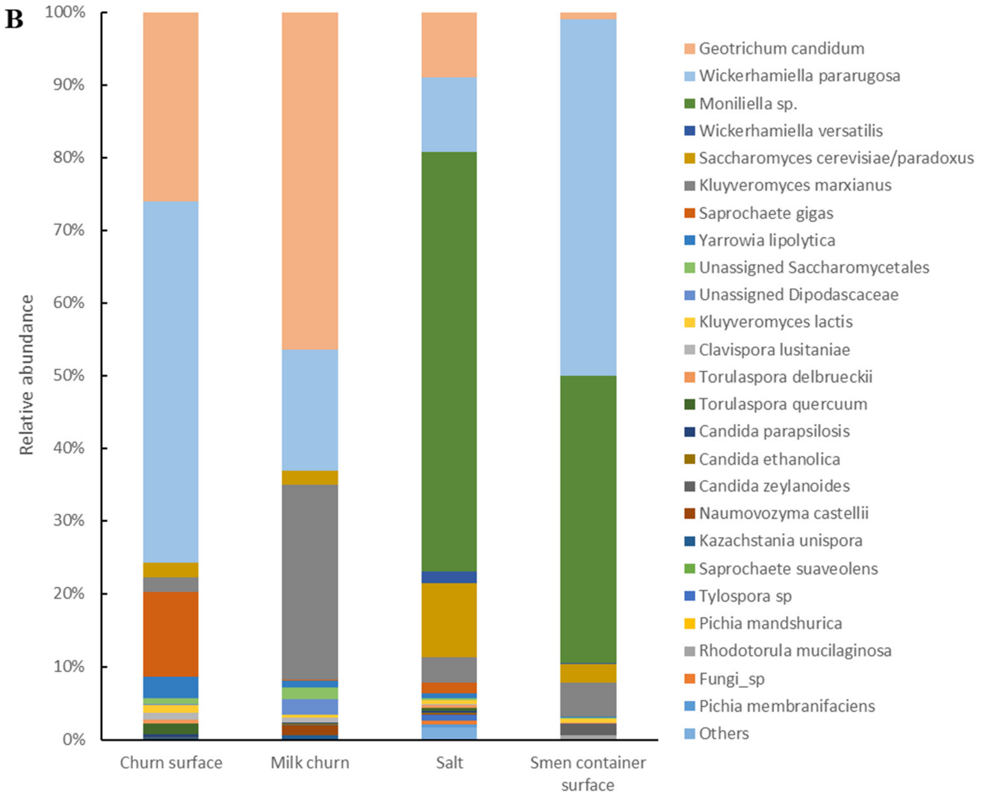

Figure S2. Bacterial (A) and fungal (B) diversity in potent microbial reservoirs encountered during *Smen* manufacturing revealed using a metagenetic approach.

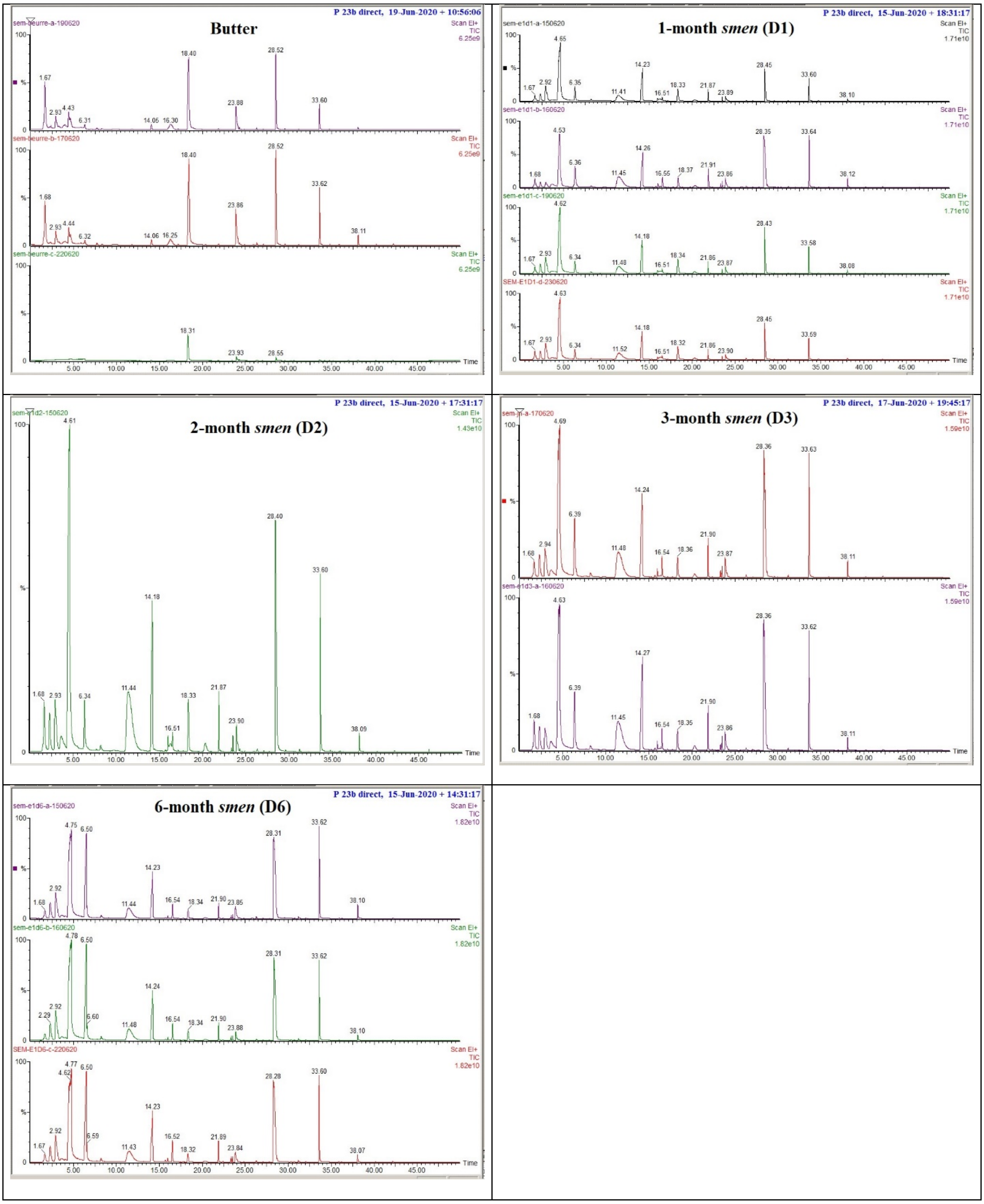

Figure S3. Examples of chromatograms obtained using headspace (HS) gas chromatography-mass spectrometry (GC-MS) of *Smen* during maturation.

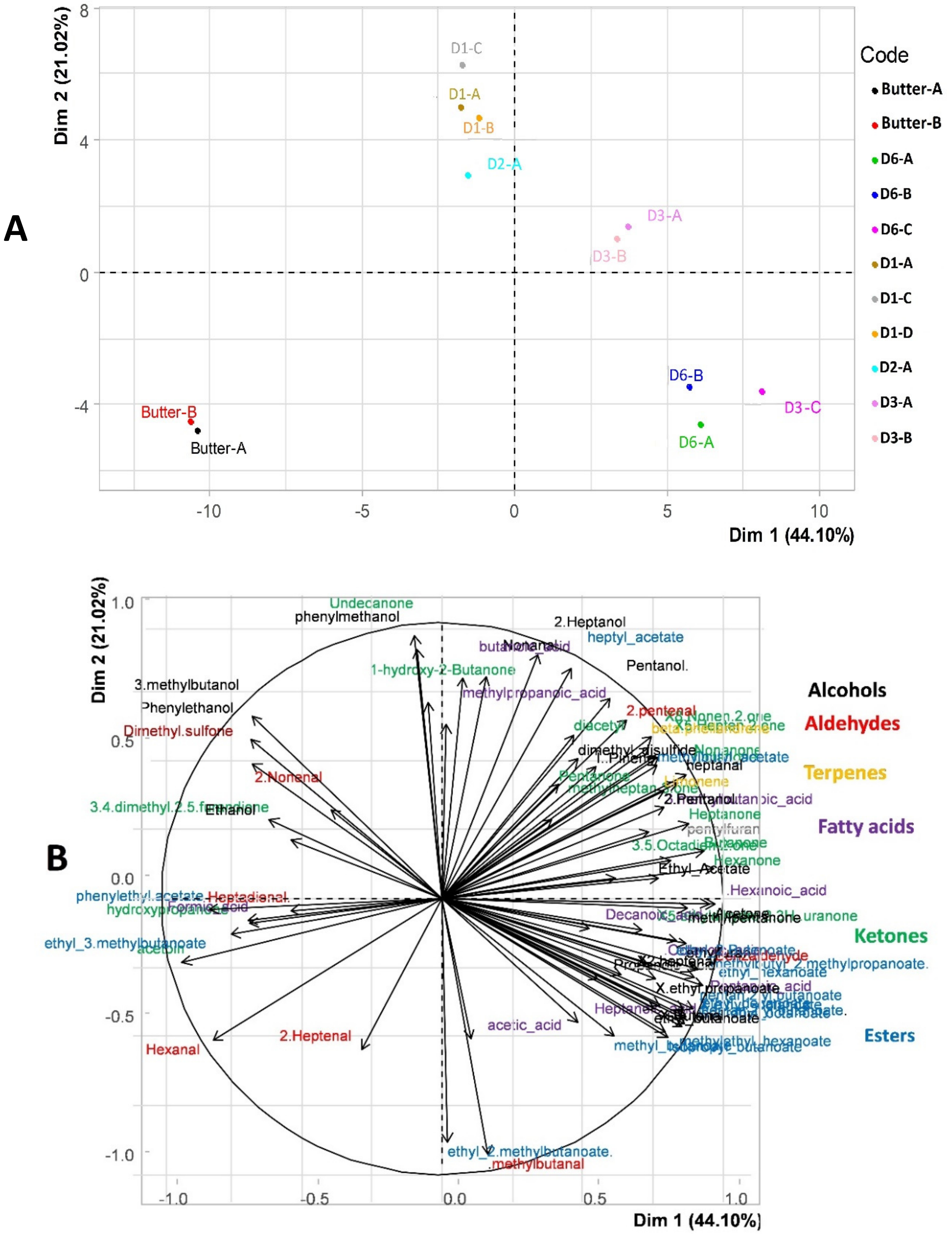

Figure S4. PCA plots of volatile compounds from the 81 volatile compounds detected throughout *Smen* maturation. PCA plot of individuals (A) and variables (B) showing principal component 1 (Dim1, 44.10%) versus 2 (Dim2, 21.02%).
